# Epidemic Cholera: An Eclectic, Miscellaneous and Clinical Review

**Published:** 1833

**Authors:** 


Art. V.—*Epidemic Cholera: An Eclectic, Miscellaneous and Clinical Review.*

Having given up a liberal portion of our last volume to the history and treatment of Malignant Cholera, we felt disposed to let the subject rest for the present, but unhappily the state of the public health commands us to resume it. The great invader seems to have slumbered through the winter, but to acquire fresh power of desolation. On the opening of spring he took the field with renovated activity; and has already made himself felt from the sunny sugar lands of the Delta of the Mississippi, over the dry and calcarious ridges of Kentucky, to the ravines and sand stone hills of western Virginia and Pennsylvania. Thus, from the sea to the mountains, through ten degrees of latitude, and a thousand feet of elevation, over a great variety of soils, and among the people of ten different states, we see the destroyer a second time put forth his strength and select his victims!

We shall not presume to attempt speculating on the causes of this resuscitation of Cholera; which seems to have taken place nearly at the same time, in several very distant parts of the valley of the Mississippi; though in others, its revival has been and still is progressive. When the cause of Cholera shall be discovered, the laws of its action, its rise and fall and reappearance, in short, its "thousand and one" anomalies and apparent caprices will be unfolded to us. How long a period is to elapse before this great discovery will be made, we shall leave it with others to prophecy. And perhaps we had better submit the whole matter to those favored members of the profession, who have already received the delicious revelation! This we should be pleased to do, if we knew which among the fovoured recipients to select. But they are not a few, and the means of determining which is the chosen vessel of Apollo, are not quite obvious. Thus, one has smelt the rising clouds of miasmata—another perceived the insensible meteoration of the atmosphere—another has heard the hum of inaudible ærial insects, or tasted the invisible animalcules of the "father of waters" and his countless tributaries—while another has perceived contagion enter at his fingers ends!—All are convinced and confident! We heartily wish ourselves of this happy fraternity; we long to ascend from the regions of anxiety and scepticism—we would believe in *something* if we could—but at present we must be excused either from believing, or from canvassing the belief of others.

It will be a more profitable employment to dedicate ourselves to the treatmentof the disease, and to this we shall proceed.

Of all the epidemics that have ever visited the nations, none has given rise to as many narratives and treatises, as Cholera. In this remote region but a small part of them reach us, yet there lie on our table no inconsiderable number, together with many Reviews and Journal essays, from some of which we propose to make such extracts as will afford our readers a diversified summary of clinical experience.

---

The first that we shall notice, is from the pen of Mr. James Kennedy, (London, 1832,) an English Surgeon, who spent some time in India, where he witnessed the disease. In *making up* his book, he has moreover, drawn largely from the sources which have furnished materials for so many other works on the same subject—the reports of the Medical Boards of Bombay, Madras and Bengal. As a practitioner, Mr. K. seems to us to be judicious. His magazine embraces but few articles, most of which are well adapted to the treatment of the disease as it appears in this country.

And here we take occasion to say, that the observations of the physicians of India, and their modes of practice, seem to us to be more valuable to the physicians of the valley of the Mississippi, than those of the medical men of Europe.

Mr. K. recognizes, in Cholera, two distinct types to which he applies the epithets *protracted* and *rapid.* The former he divides into two stages. Symptoms of the first stage.

> "*First Stage.*—The patient complains of a feeling of anxiety, or of uneasiness at the pit of the stomach. After some time nausea supervenes, and the uneasiness changes into a feeling of heat or pain. To these symptoms succeed vomiting and purging, and prostration of strength. The evacuations, at first, consist of the common contents of the alimentary canal; afterwards, of a fluid like rice-water. Occasional cramps are felt in the limbs. The pulse is small, and rather quick. The skin feels a little cold, and the temperature is gradually decreasing. The countenance of the patient is somewhat shrunk, and the features appear sharper than natural.
>
> If the disease be left to itself, or if it continue to advance in spite of the remedies that may have been used, the symptoms increase in severity, and the patient comes to suffer from
>
> Violent cramps in the upper and lower limbs, and at times in the muscles of the chest and belly. The cramps, in general, are not constant; they recur at short intervals in paroxysms. The vomiting and purging are severe. The coldness of the skin has increased much; it feels moist, and is of a bluish colour about the face, hands, and feet. The palms and soles of the latter appear corrugated, as if they had been steeped in water. The pulse is barely, or not at all to be detected in the wrists and temples. The countenance is ghastly, and expressive of great anxiety. There is distressing thirst, or burning heat or pain in the region of the stomach and bowels.
>
> If the disease be still uncontroulled, it will pass into the second stage.
>
> *Second Stage.*—Under the increasing debility, the vomiting, purging, and cramps are subsiding, or have disappeared. The patient lies in a state of helpless exhaustion, and is almost incapable of making the slightest movement. He is apparently insensible; but as his senses remain unimpaired to the last, he may be roused to say "Yes," or "No." The pulse is gone, and even the action of the heart is extremely feeble. The surface of the body is deadly cold. The breathing is suppressed, or scarcely perceptible, and the countenance is quite cadaverous."

The American reader, who has seen the Epidemic, must be struck with the identity of the symptoms here enumerated, with those presented by the disease in this country, except in the absence of the initial diarrhœa, so common in the west, which, however, is often present in Asia.

"In the treatment of the first stage, the primary object of the physician will be to allay the cramps, the vomiting, and the purging. To carry this indication into effect, he should not rest content with the application of any single remedy; it will be accomplished most effectually and rapidly by combination of measures. The warm bath, blood-letting, and a large dose of calomel and laudanum are to be immediately prescribed. If tbe bath be ready prepared, the patient should be placed in it, and during immersion he may take the dose of medicine, and have the blood-letting performed. One remedy, however, should never wait upon another; the first at hand should be administered first. Their influence upon the symptoms is to be vigilantly watched. As soon as the cramps are subdued, or have received a decided check, the patient with all possible expedition, should be removed from the bath, and placed between *dry* heated blankets. Dry warmth should be further afforded by surrounding his body and limbs with bags of heated sand. If the cramps, &c. do not return, the previous remedies are not to be repeated. New measures are now required to remove debility, and to excite the natural action of the bowels.

The treatment of the *second* stage will be very different from that of the preceding. During the active progress of the first stage, the vital power is considerable, and there is high spasmodic action in various parts of the body, which enable the patient to bear with advantage the loss of blood and the sedative effects of large doses of laudanum; but in the second stage, the patient is in a state of *real* exhaustion, and would consequently sink under the use of these strong measures. Even the warm bath is dangerous to life at this period, and its use must therefore be avoided.

In the second stage there are two principal indications for simultaneous adoption—1st, To remove the deadly coldness of the body; 2nd, To remove the great debility. The coldness must be attacked by the application of *dry* heat. The bags of hot sand should be assiduously applied to every part of the body, and in addition, heated blankets above and below the patient. For the removal of the excessive debility, some of the most powerful stimulants must be had recourse to, as ether, brandy, or other spirits, hartshorn, &c. These remedies are to be continued until the patient finally sinks under the disease, or until reaction is fairly come round, after which they may be gradually dispensed with."

The length of time which elapses in different cases, while the disease is passing from the first to the second stage, is extremely various. When it is *very* short, the case partakes in a corresponding degree, of the *rapid* type, and, consequently the two must be regarded as differing only in the grade of malignity. The *symptoms*, however, are found to be widely different when we compare the ordinary examples of the two different types.

"RAPID TYPE.—In many instances the attack of cholera commences with very aggravated symptoms: the patient falls suddenly down in a state of extreme weakness, and perhaps temporary insensibility, and the energies of the constitution are so much oppressed by the influence of the morbid poison, that there may be little or no visible attempt at reaction. The pulse almost instantly, or in an exceedingly short period, disappears at the wrist, and the heat of the body rapidly declines. In these cases, cramps, vomiting, and purging, are either completely absent, or an attempt to establish these symptoms is only evinced in slight spasms, and the occurrence of one or two nearly passive evacuations."

During the Epidemic of 1832, in this city, but a few cases of this kind occurred. We have lately witnessed one nearly of this character; and many have, it is said, occurred in Lexington and other parts of the state of Kentucky in localities generally of the healthiest kind.

Our author divides the rapid type into two stages one of *opparent* the other of *real debility*—oppression and exhaustion.

"The chief remedial measure is blood-letting; and here its use is founded upon the fact, that, for a certain space of time after the commencement of the attack, the resources of the constitution are not to be measured by the external symptoms. Apparently the patient is in a state of hopeless debility; (and this will generally prove to be the case, if he be left to the efforts of nature alone;) but when properly assisted by art, the constitution is often enabled to rally, and eventually to recover from the shock. The time, however, in which this medical service may be performed with the greatest hope of success, is unfortunately extremely limited, in some cases not exceeding many minutes after the patient has been seized.

If the practitioner arrive before the blood has deserted the superficial vessels, blood-letting should be performed to the extent of twenty or thirty ounces, in an adult. Then other auxiliary measures will be required. Of these, nothing should supercede the immediate and continued application of dry heat to the surface of the body. The value of this remedy is incalculable, and it has the advantage of being applicable at any period, whereas the opportunity for the abstraction of blood may be soon irrevocably lost. Internal stimulants, as ether, hartshorn, &c. should be also administered, in order to expedite the establishment of reaction. If the second stage, that of real debility, have commenced before the patient is seen, the treatment will consist of dry heat and internal stimulants, without blood-letting.

"In many cases of the rapid type, a complete inversion of the train of symptoms common to the protracted variety of cholera, has been observed during the progress of reaction. As a patient recovers from the stateof stupefaction, or apparent exhaustion, into which he was hastily precipitated, and as he graduallyg athers strength, it may happen that cramps, vomiting, and purging, will then be developed in the first time. This proves, as it were, by analytic evidence, that cramps indicate a comparatively mild form of the disease. If the cramps become severe under this new arrangement of the morbid phenomena, we must resort to the curative means hitherto recommended for the spasmodic stage and its consequences."

On the subject of the warm bath in this type of Cholera ,our author has the following observations, which we recommend to our practical readers.

"In the abstracts of the Indian Reports, instances may be observed where the use of the warm bath in the second stages of acute cholera seemed to hasten the death of the patients. Instances of a similar character might be multiplied by referring to facts extraneous to this volume, but a few will be sufficient for our present purpose. That moist heat should have a dangerous influence on patients in a state of *real* debility, appears very natural, when it is recollected that even persons in health will faint* after long immersion in a bath at a high temperature. In the treatment of cholera, the temperature of the bath was generally above 100 degrees, and frequently was raised to 110 degrees, in the hope that this strong heat might counteract the rapid loss of the natural warmth of the body. In our opinion, the warm bath, or any other form of moist heat, should never be used in acute cholera, unless at the commencement, or during the prevalence, of severe spasmodic symptoms."

Mr. Kennedy is no great friend to opium in Cholera. He thought it much abused in India. It was often given in large doses throughout all stages of the disease, with or without brandy, and very often he thinks did harm. In the first stage of the protracted type, combined with calomel, he found large doses beneficial, and thinks the utility of that powerful narcotic nearly limited to that stage. We do not entertain a doubt that opium is far more benificial in the early than the latter stages of Cholera.

Our author's views in regard to blood-letting in cholera meet

* "If a patient in acute cholera be placed, daring the stage of real debility, in a warm bath, he will probably faint, and never recover from the fainting fit thus induced."

our entire approbation. He first considers the circumstances that contraindicate it. Thus it should be avoided—first, towards the termination of the first stage of the protracted type; second, during the whole of the second stage; third, in the rapid type during the period of debility. In general, when blood-letting would be improper, such is the state of the circulation, that blood will not flow, but there are exceptions to this rule.

"Blood-letting, the opportunity for performing which is so precarious, is not indispensable to the recovery of a number of patients who are subjected to cholera in either type; but patients who have been freely bled, in general recover more rapidly than those who have not. The blood-letting appears to cut short the attack in a decided manner, and afterwards, with the establishment of reaction, the secretions which had been suppressed, the heat of the skin, the pulse, and the appearance of the patient, gradually return to their healthy standard. In cases where recovery is slow, and more particularly in such as have not been bled, reaction is occasionally the forerunner of febrile symptoms and derangements of the bowels, which impede the progress of the cure, and require to be treated on the general principles of medicine."

Mr. Preston, of Madras, has given phosphorus in the stage of "real debility," (exhaustion) and entertains a very favorable opinion of its powers. It is an exceedingly strong stimulant. He has reported two cases in which it was decidedly successful. We should think it deserving of attention, if indeed any thing can be said to merit a trial, in the mortal stage which it is designed to combat.

Next follow remarks on emetics, blisters, magnesia, castor oil, alkalies and acids, none of which, in the opinion of our author, deserve much confidence, and should never be substituted for blood-letting, laudanum, calomel, the common stimulants, and moist or dry heat, as the stage or type of the malady may require.

Dr. Spencer, of the state of New York, in a communication to the Medical Society of that state, February, 1833, has in reference to the first stage of Cholera the following propositions:

"1. The epidemic influence being thrown upon the exhalent tissue of the small intestines, renders it highly susceptible to the action of irritants, so that the imperfectly digested food or mild laxatives often excite profuse evacuations, and there is thus produced a disposition to violent disease from the common exciting causes of diarrhœa.

2. The stomach being weakened in its function, digestion is imperfectly performed, and at times almost suspended.

3. The liver responding to the stomach from its habitual sympathetic relations with that organ in health, falls into a state of torpor, and bile is no longer secreted."

Dr. Spencer lays great stress, in his pathological views, on the irritation, increased determination to, and augmented discharge from, the exhalent tissue of the mucous membrane. It often amounts to quarts, and, according to our author, even gallons in a few hours. He compares it, as to its origin and effects, to mucous hæmorrhage. The chief difference, in his opinion, lies in the loss of the serum of the blood in one case, and the serum and red globules in the other. From this loss he deduces the stage of collapse—a state of suddenly induced vascular inanition. Let him speak for himself.

"We have now examined every source but one, without finding any from which these large serous discharges can be derived. The only one remaining is the exhalent tissue of the intestines, and it is believed we may now draw the inference that the exhalents are the floodgates by which the vital fluids are thus rapidly drained from the body. If, then, these vessels give exit to the discharges, may we not infer that the *exhalent tissue of the bowels is the proximate seat of disease in the second stage*, as well as in the first, as already proved. In the exhalents, then, we have a natural outlet to these fluids, and of course should expect no lesion of the membranes when the patient died in collapse—a *fact* most fully proved by the universal testimony of the profession, who have made a record of their dissections, throughout the globe. It is believed that the *essential phenomena yet to be accounted for, can be traced to these discharges as a cause*, and if so, the conclusion seems irresistable that we have correctly arrived at the proximate seat of disease. If, then, the proximate seat of disease is the same as in the first stage, when all will admit the disease is serious diarrhœa, shall we change the name and call it "cholera," because an indigestible meal or a debauch greatly increases these serous discharges, and vomiting supervenes? In what has preceded, we have noticed only the serous evacuations, while the *colour* has not been accounted for. The first effects which would naturally result from these discharges would be to wash out whatever might be in the alimentary canal at their commencement. At times these are yellow, or they look like *dirty water*, throughout the disease, and the discharges in these cases have a feculent smell. Dissections have shown about five cases in one hundred in which the colon was distended with feculent matter in those who have died of this disease, and this circumstance rationally accounts for such occasional appearance of the discharges. If part of the food remains upon the stomach, or the chyle remains unabsorbed, this gives colour to the evacuations, which sometimes resemble milk and water, or thin gruel. The follicular glands at times, on dissection have been found to exude a milky fluid, which may likewise give this or the rice-colour to the serous discharges. Rice-coloured evacuations are often observable in the chronic intestinal diseases of children. The red tinge seen at times in the latter stages of this disease, and in the intestinal contents on dissection, must result from the admixture of red globules of blood with the serum of the discharges. *Colour* would, therefore, appear as an *accidental* circumstance, while the serum of the discharges is *uniform*, and in a large proportion of cases almost colourless.* When the pathological relations to other diarrhœal affections are traced, serous discharges will be found to constitute the source of danger in all."

Our author proceeds to inquire, *seriatim*, into the effects of this profuse exhalation, upon the mass of circulating fluids, the heart and vessels, the respiration, the capillary functions, the secretions and evacuations, the absorbent system, and the functions of animal life. Our limits do not permit us to follow him through his ingenious reasonings on these different points, and we shall proceed at once to his—

"*Recapitulation.*—From a review of what has been advanced on the nature of the second stage, its essential pathology may be summed up in a few propositions.

1. The disease essentially consists in this stage, in a determination of fluids to the inner surface of the small intestines, diverting the respiratory, perspiratory, and urinous discharges, with their natural salts, from their usual channels; *and discharging them through the intestinal exhalents*, rapidly emptying the blood vessels of their contents, and changing the relative proportions of the remnant of circulating fluids.

2. That the failure of the functions of the heart, lungs, capillary cir-

*Scott.

culation, and various secretions, results from direct depletion, depriving those organs of their accustomed stimulus.

3. The absorbent system is rapidly taking up the adipose and waste parts of the body, to supply the failing resources of the heart, and thus results the rapid emaciation.

4. The spasms of the voluntary muscles, and those drawn into contractions in the act of vomiting, by compressing the intestinal exhalents, tend to arrest the discharges; and by aiding the return of the venous circulation, stimulate the heart to redoubled exertion, giving a centrifugal direction to the circulation, thereby making a metastasis of the exhalation from the inner surface of the bowels to the skin.

5. That a striking analogy exists between this disease and hemorrhage, differing only in its effects upon the constitution, from the circumstance of its changing the relative proportion of the ingredients of the blood."

These views are highly plausible, but they are met by the unquestionable fact, that many cases prove fatal, in which the discharges are by no means profuse. Such must have occurred in the practice of every physician. We have lately met with two, in which the patients passed rapidly into fatal collapse, with inconsiderable evacuations from the stomach and bowels, and without any distension of the abdomen, that could suggest that much fluid was accumulated in those viscera. Indeed, we are convinced, that the primary impression of the remote cause, is made upon some part of the nervous system, and that the great infiltration of serum into the bowels, is a secondary affection which, however, may, when it occurs, be the cause of much that he has assigned to it.

Dr. S. entertains just views of the relation between epidemic and endemic Cholera. We shall transcribe his summary of the symptoms which respectively characterize them:

| | |
|---|---|
| "The epidemic almost universally commences with diarrhœa, of several hours or days' continuance. | Cholera morbus is scarcely ever preceded by diarrhœa. |
| The commencing diarrhœa of the epidemic is devoid of bile. | If diarrhœa precedes cholera morbus it is generally bilious. |

| | |
|---|---|
| Water is the principal discharge in the early period of the epidemic. | Bilious matter is the principal discharge of cholera in its early stage. |
| If bilious discharges come on in the epidemic, the patient is considered safe. | If the bilious discharges cease in common cholera and watery stools come on, there is great danger. |
| The epidemic as well as the common diarrhœa is a disease of all climates and all seasons. | Cholera morbus is peculiarly a disease of hot weather, and warm climates. |
| Vomiting is by no means a uniform symptom of the epidemic. | In common cholera, vomiting is a uniform symptom, when absent called bilious diarrhœa. |
| The epidemic is always greatly aggravated by changes of weather from warm to cold. | Cholera morbus is increased by hot sultry weather supervening upon cool. |
| In the epidemic, the hepatic function is suspended from the first dawnings of the disease. | In common cholera, the liver is actively excited and the acrid bilious matter secreted becomes the exciting cause of the disease." |

The ninth chapter of our author's work relates to the treatment of the first or diarrhœal stage. His instructions are, in general, judicious; but as we have, in former numbers, entered fully into this subject, we shall not dwell upon them. His opinions in regard to drinking, correspond with those already put forth in this journal:

"Notwithstanding the high authority by which brandy has been recommended as a preventative of the attacks of this disease, I am fully satisfied, that ardent spirits are not only unnecessary, but injurious to those previously unaccustomed to their use. To those, however, habituated to its daily employment, it may be proper to say, that a sudden suspension of the habit during the epidemic, might expose them to hazard, but great moderation should be exercised, since the slightest excess is liable to bring on the severe grades of the malady. Malt liquors, in moderate quantity, from the carbonic acid they contain, afford a grateful stimulus to the stomach, and aid the process of digestion. Port or Madeira wine may be advantageously employed for the like purpose, when the function of the stomach is weakened from previous disease, or when great oppression is produced by the effects of the epidemic influence. Under such circumstances, wine produces a temporary aid to digestion, especially if a meal remains long unchanged upon the stomach."

We cannot too often or too strongly, impress on our readers and the public generally, the necessity of attending to the first stage of Cholera. All experience has shown, that it is, then, a disease which is easily arrested, while, if suffered to pass beyond that stage, it becomes one of the most unmanageable. The moral power of the profession should be put forth on this point. Every physician should feel it a sacred duty, to raise his voice against the neglect of early symptoms. He may save many lives without making one prescription, by warning his patients of the necessity of doing something and refraining from many things, when they first feel indisposed.

Our author condemns purging in the early stages of Cholera, a prohibition in which we entirely concur, unless it be effected by calomel or the blue pill or rhubarb conjoined with ipecacuanha. Even then, it is not the *purgative* effect of these medicines, on which their efficacy depends; but their action on the liver, the function of which is impaired or suspended, and must be restored, or the disease will advance and the patient die.

In the treatment of this stage of the disease, our author lays deserved stress on the use of emetics. They should act promptly and efficiently. Tartar emetic from its action on the bowels, is not so proper as ipecacuanha and the sulphate of zinc.

"This occasional effect of emetic tartar renders it somewhat objectionable, and it is believed that the sulphate of zinc and ipecac, as recommended by Dr. M'Naugton, may be safely and advantageously substituted in most cases, where the diarrhœa has been of considerable standing. Sulphate of zinc 5 grs., combined with 20 or 30 grs., of ipecac, may be given in a wine glass of warm water, and if not vomited in 8 or 10 minutes may be repeated, or 20 grs. of ipecac alone may be repeated every few minutes till full vomiting is produced. The eupatorium, from its emetic, diaphoretic, and tonic properties, is admirably adapted to the cure of this disease, and a strong infusion of this article or chamomile may be very advantageously administered to promote the operations of the emetics."

We have lately been informed, that in the recent visitation at Georgetown, Kentucky, the salt and mustard emetic of the Hindoo practitioners, was used with admirable effect. It was given in anticipation of the compound of calomel and opium, which was found so beneficial in this city, in the first stages of the epidemic last autumn.

Chapter 10th embraces the treatment of the second stage. The leading indications are—

"1. To arrest the intestinal discharges.

2. To make a transfer or metastasis of the serous discharges from the exhalents of the bowels to those of the external surface.

3. To restore the lost balance and healthy performance of the various excretory and secretory functions.

4. To support the powers of the system, and combat incidental symptoms.

The first three indications may in a large proportion of cases be simultaneously fulfilled by a combination of active emetics and sudorifics. To these the disease yields with great promptitude, provided a dose sufficient to act efficiently is employed. Nothing can, however, be more improper than the exhibition of half doses of emetics in this stage, since every thing depends upon making a prompt and decided impression upon the disease. If the operation of emetics is prompt and active, all the energies of the system seem to be aroused —the face resumes its wonted flush, a free warm perspiration starts from every pore, and the patient is comparatively safe."

One of our author's friends, Dr. Benjamin Palmer, for the purpose of arresting the profuse alvine evacuations, or of obviating their effects on the circulation, resorted to ligatures to the extremities, as in cases of hæmorrhage, and found them serviceable.

The following extract will present our author's views of blood-letting in Cholera:—

"If the pathological views of the disease presented be correct, bleeding may find an appropriate place among our remediate means, but its indiscriminate use must be exceedingly hazardous. The operation of this remedy in the epidemic, must be very similar to its action in hæmorrhage, and if employed before the action of the heart and arteries is prostrated by the severe discharges, I can discover no good reason why it is not equally safe to unload the blood vessels by the lancet, as to have them thus rapidly emptied by a continuance of the large serous evacuations. It is thought that by bleeding the patient from a large orifice, until faintness is induced, the discharges may be thus stayed, and by directing our remedies to the restoration of the various secretions, the patient may be cured.

Some medical practitioners have very successfully treated the disease in this manner, but it must be obvious that the remedy is applicable only to the early part of this stage, when the pulse remains vigorous; a period which often passes before professional aid is called. If the pulse has, however, failed, and the system is rapidly approaching the collapse, or this actually exists, no good reason can be discovered for using blood-letting in this disease, any more than in any other malady, when the vital enegies have been prostrated by direct depletion. If we found a man nearly pulseless, from natural hæmorrhage, or from the division of any artery, we should hardly be considered rational, if we undertook to bleed him to arouse the system. Now, it is thought equally proper in this case, as in the collapse of the epidemic, when, as frequently happens in rather slow cases, the patient has already lost a quantity of fluids, equal in volume to the estimated amount of blood ordinarily circulating in the heart and blood vessels. Those cases which occasionally occur in this epidemic, and which often happen at the onset of febrile diseases, in which extreme coldness results, from an oppression of the sanguiferous system, resulting from a congestion of the fluids, in the internal organs, unattended with evacuations, are often benefitted by bleeding. But this collapse should be carefully distinguished from that resulting from large evacuations, the principles of treatment being widely different."

Dr. Spencer seems to attach but little importance to the powers of calomel in this malady. The liver in his opinion is secondarily affected. It is the copious exhalation from the bowels that has suspended the action of the liver. But, admitting the accuracy of this *speculotion*, may not calomel do good? If an excited state of the mucous membrane can divert *from* the liver, may not an excited state of the latter, attract the blood from the former? He seems to be afraid of the *purgative* effects of calomel; but we have not witnessed them, and if apt to occur, might they not be prevented by the addition of opium?

"Given in minute doses, however, it sometimes acts beneficially in allaying irritability of the stomach, and exciting a healthy, peristaltic motion of the bowels."

Our own experience does not warrant a preference for minute doses of this medicine. On the contrary, we have found large doses, even 30 or 40 grains, at more distant periods, decidedly preferable. They subdue the irritability of the stomach and bowels, more successfully than small portions; and, instead of purging, not unfrequently suspend the diarrhœa, by allaying the irritation which excites it.

The 11th chapter is devoted to the stage of collapse. The indications are:—

"1. To restore to the heart and blood vessels the stimulus of distention, by supplying them with the fluids, and neutral salts, of which they have been deprived.

2. To sustain the sinking powers of life, and combat accidental symptoms."

Our author's pathological views make him a decided advocate for the liberal use of water, and incline him to the saline practice recommended by Dr. Stevens, of the Island of St. Thomas, in the fevers of that place, and afterwards for Cholera in London. After stating that gentleman's recipe, which we have already laid before our readers in a former number, and shall presently repeat, he gives another, suggested and, it is said, used with advantage, by Dr. Harris, of Philadelphia, which we shall transcribe.

℞. Super-Carb. sod. ʒ1.

Mur: sod. ℈1.

Chlor: pot. 8 gr., mix.

"A simple solution of common salt, or combined with the other neutral salts, with starch, as the vehicle, may be used as an enema, which should be drawn off with a tube, and repeated at like intervals. Should they tend to run off, they should be retained by the means recommended in the second stage. If, however, these neutral salts are found to keep up or augment the serous discharges from the bowels, they are obviously contra-indicated, and aqueous fluids alone, or slightly impregnated with nitric acid, as advised by Mr. Annesly, or combined with camphorated spirits, should be used. Even these ought likewise to be employed according to the effect they produce, requiring at times to be sparingly used, or their employment suspended, as in the second stage, when the stomach and bowels need to be tranquilized."

Dr. Spencer seems to lean to the practice of injections into the veins in this state of Cholera, and gives some reasons why as he thinks, they have not been successful. He suggests that the fluid should be thrown in very gradually; and intimates that simple water might perhaps be safer than a saline solution and equally beneficial.

Opium, in the opinion of our author, is but little indicated in this stage, and often proves injurious. Among other bad effects he supposes it may contribute to diminish the secretion of urine.

We need not dwell longer on the treatment of the state of collapse. Like most other physicians, our author seems to have but little confidence in any thing yet proposed. We believe the stage of confirmed collapse or approaching death, to be as little and as much, under the controul of medicine in this disease, as in any other. When preceded by copious evacuation, it is perhaps, scarcely ever cured. When suddenly induced, it is more manageable, and should, as we think, be treated with a stimulating emetic, venesection, and the partial and sudden application of ice or iced water.

The last chapter is devoted to the nature and treatment of the consecutive fever. We shall dwell upon it no longer than to make the following extract:

"The urinary secretion being generally suspended in this fever, it becomes a matter of paramount importance to restore it, or fatal coma too frequently supervenes. By combining these three articles, we have one of the most active anti-inflammatory combinations. A powder of calomel, 1 or 2 grains emetic tartar, ¼ gr. cream tartar, 8 or 10 grs. may be given every two or three hours, at the same time spts. nitre and mucilages are employed."

---

We open another New York Cholera book, consisting of a series of letters from Dr. Martin Paine to Professor Warren, of Boston, written in 1832, while the epidemie prevailed in the former. As might be expected, it is of a desultory character, and we shall not attempt its analysis.

As every fact relative to the first stage of Cholera is of paramount importance, we copy the following:—

"Although the predispostion to this epidemic is generally developed by some of the premonitory symptoms which I have indicated, it occasionally happens that the attack is very insiduous. The tongue may be clean, and of a healthy appearance: the evacuations at first bilious, and unattended with pain; perhaps little or no vomiting and no evidence of danger in the countenance; yet I have known a sudden transition of these auspicious appearances to an array of the most malignant symptoms.

I have said but little in respect to the state of the tongue as an index of the disease, and still less of the pulse, and skin, and secretion of urine. They are so variously affected, under apparently the same circumstances, that they cannot be assumed as criteria. The tact of the physician, therefore, must convert them to the best advantage in each individual case."

Dr. Paine's report of the efficacy of calomel differs widely from that of Dr. Spencer, whose work we have just reviewed, and seems to us so correct and important that we are tempted to place it before our readers. It corresponds most strikingly with our own experience, and that of the physicians of Cincinnati generally.

"In regard to *calomel*, my own experience is sustained by that of our best physicians. From the particular irritability of the mucous membrane, and the profuseness of the discharges, the prepossessions of many physicians, who now regard it with a sense of dependence, were adverse to its exhibition. The little encouragement, however, which they derived from other remedial agents, directed their attention to the declaration of foreign physicians, that this medicine not only allays the irritability of the mucous membrane, but induces that change of action which is necessary to the re-establishment of health. I may safely assure you, Sir, that our best practitioners now employ calomel in doses of 10 to 30 grains, with more confidence than they repose in all other remedies. Either with or without small quantities of opium and camphor, according to the existing state of the serous evacuations, it seldom fails to allay the irritabilty of the mucous membrane, and of the system generally; to mitigate or remove the spasms; and if the case be susceptible of relief, to convert the discharges from the appearance of rice-water to that of the green confervæ, or the not infrequent blackness and consistence of boiling tar. This transition is nearly a sure omen of a radical change through the whole system."

Dr. Rowe of the Greenwich Cholera hospital, used with much advantage an ointment composed of 1 pound of strong mercurial ointment, 7 ounces of Cayenne pepper and 7 ounces of camphor, applied over the whole surface of the body, with a brush, and frequently repeated till reaction or a salivation took place.

"Another ointment has been compounded by Dr. Rhinelander, of the Crosby-street Hospital, and is prepared in the following manner: Take of red pepper and camphor each half a pound, simple ointment one pound, muriatic acid one ounce. Triturate together the powdered camphor and pepper, adding the acid. Pour the mixture into the melted ointment, carefully stirring it. The ointment is very freely applied to the whole surface, and the skin afterwards covered with hot chalk. In this preparation, the active principle of the pepper is evolved by the acid and it possesses, therefore, the advantage of being more stimulating than the other, but it wants the specific property which is imparted by the mercury."

Dr. P. condemns the hot bath. He observed an extraordinary epigastric heat. In one case the thermometer rose to 106 degrees. He thinks ice to that part and to the muscles affected with cramps, a better application than mustard. He disapproves of high internal stimulation and of much opium, prefering the sulphate of morphia to that drug. Dr. Caldwell of Montreal, in a letter to our author, informed him:—

"That he employed as a cathartic, after the exhibition of calomel in the earlier stages of this disease, tart. potassæ ℨij. in a little warm chicken broth, which he repeated every second hour until it operated freely. "Nothing," says that eminent gentleman, "seemed to allay the gastric irritation so well as the solable tartar. In many instances it seemed to act as a charm, and I do not know a single instance of any one, who went through the influence of this treatment, that was subsequently attacked with Cholera."

Our author of course denies to camphor any specific power, but thinks it a useful adjuvant. He tried tobacco injections in two cases, as practised by Mr. Baird, of England, but found them injurious. In two others, he knew of the use of injections of a strong solution of alum (*Sup. sulph. alum, et pot.*) with no better effect.

"*Cupping* and *leeching* have had a full proportion of our attention. They are less salutary than general blood letting, when it is an object to produce an impression on the whole system, or for the relief of high vascular action, whether local or general. In Asphyxiated Cholera, where there exists more than the usual determination of blood to the head, they have been found useful, and contribute to the relief of oppression at the precordia, and some of the varieties of abdominal distress. Like general blood letting, the local abstraction is often indispensable, in the treatment of some of the consecutive diseases."

Venous injection was tried and found unsuccessful. It was employed in numerous instances in the hospitals and in private practice. So of inhalations.

"The *oxygene* and *nitrous oxide gases* have been tried at the Greenwich and other hospitals; but I am informed by the gentlemen connected with three of those institutions, that they have obtained no benefit from their use,—and that the blueness of the skin was not even influenced."

Dr. Paine's letters contain a variety of speculations on the pathology, and many details on the morbid anatomy of Cholera, which, intending this as a clinical review, we shall not attempt to lay before our readers. Among the latter, however, we find one which seems new, and deserves to be recorded on our pages. It is the presence of oil in the blood, which was observed in several instances. It was first noticed by Dr Gale, who thus speaks of it, in an athletic and intemperate negro, who died in one of the Cholera hospitals of New York.

"The oil, to which I have adverted, was remarkably abundant in this subject. It covered the blood from whatever part abstracted, and floated in small and large globules on the surface of about one ounce after standing an hour in a vessel, into which it had been received from the heart. There were two globules of the size of a common pea; the others were very numerous, but no larger than a grain of mustard. It was found in the large and small vessels—it was very distinct in the brain—it was as obvious in the kidneys, and in every part particularly examined. It resembles olive oil in appearance and consistence."

This oil was doubtless absorbed from the adipose tissue during the progress of the disease.

---

In a pamphlet on the history and treatment of the Epidemic, as it appeared at Vienna in 1831 and 1832, Mr. J. F. Fergus, a British surgeon, has communicated to the profession a neat expose of its symptoms and the method of cure found most successful. We shall take no notice of the former, except as connected with the latter. On the exciting causes of Cholera, Mr. Fergus has a variety of remarks. The following facts are worthy of being recorded.

"Fear is another exciting cause of the greatest force. A Dr. Bochie was sent into Hungary to treat the cholera, where he was in a short time attacked, and died. He had left a mistress at Vienna. A brother of the deceased, meeting the girl in the street, said, "Your friend has died of the cholera." The girl was much shocked, and before she reached her home she was seized with violent cholera, and died in six hours.

Going into the hospital one day, I found a strong healthy woman, who had been at the moment brought in. She had just vomited a large quantity of half digested flesh. On asking her to what she attributed her illness, she said that her master had a friend who had been taken to the cholera hospital, and that he had sent her thither to inquire how he was. As she was going along she was in great fear she would catch the cholera at the hospital; but long before she came near it, she felt a sudden giddiness, became faint, and vomited. This proved a very severe case of the first form of cholera."

In Europe, men are much more separated into castes or classes, than in America, where the differences are often almost nominal.

"Galizia is inhabited by Poles and Russneaken, Jews and German colonists. The Poles and Russneaken, especially the latter, are very badly fed and lodged. They seldom taste beef or mutton, living mostly on a coarse rye bread, cheese, butter, milk, and sometimes a little fat bacon. They live with their cattle, under one roof, only separated from them by a partition. They are very dirty in their houses and persons, and are generally surfs. The Jews are still more filthy than their neighbors the Poles; their houses are much more overcrowded. But they are generally warmly clothed, and otherwise have more of the comforts of life. They are generally employed in commerce.

The German colonists are clean, and live much better in every respect than any of the others; they are occupied principally with the arts, commerce and farming. These three people live in separate villages, but which are often so close together as to form but one town, or "marktfleck," as it is called. When the cholera broke out, about one in every eight was seized amongst the Poles and Russneaken; about one in every forty amongst the Germans. The Jews, according to circumstances, approached more to this or to that extreme.

The proportion of deaths in those taken ill, remained always much the same. At least the difference in favour of the Germans was not very marked."

The following are the forms or divisions of Cholera, which our author observed at Vienna. Each required a different treatment from the other.

"1. The highest degree:—Seizure sudden; evacuations almost or altogether wanting; cramps most severe; ends in most cases in a few hours, the action of the heart often becoming imperceptible before consciousness is lost.

2. The most common form:—It presents the greatest variations, and the most numerous succession of symptoms, which at one time resemble more those of the 1st, and at others more those of the 3d form. Almost always a prodrome; ends fatally by paralysis, the consciousness being lost; respiration the last vital function which ceases.

3. Often no prodrome; comes on with sudden and copious evacuations. The patient sinks rapidly and silently, without any violent symptoms."

The style of Mr. Fergus is so compact, that we shall not attempt to abridge what he says on the treatment.

"*Treatment of First Form.*—In the most violent cases of the *first form*, all treatment whatever was found unavailing. Bleeding always produced relief, and if, by means of ipecacuanha, or other emetics, or by ice, vomiting was produced, the patient immediately found himself much better. But, in most cases, it was impossible to produce vomiting by any means, and the patients were all lost.

If those symptoms, described as the *prodrome* of cholera, had come on in consequence of exposure to wet or cold, the best effects were seen, if the patient went into a warm bed, took a dose of Dover's powder, and drank an infusion of elder or chamomile flowers, until full perspiration was produced. If they had been caused by an excess at the table, if nausea came on, if the diarrhœa became very frequent or copious, with cold extremities or cramps, the greatest advantage was obtained from an emetic of ipecacuanha; afterwards an infusion of the same root, 1 scruple to 6 ounces of water, with 10 or 20 drops of laudanum, a table-spoonful to be taken every hour. In a day or two, when the purging still continued, the infusion of columba was substituted for that of ipecacuanha, and the quantity of laudanum increased. Under such treatment it was very rare that these symptoms went further.

*Treatment of Second Form.*—If the patient was strong, the very best effects were seen from bleeding; and then the administration of a large dose of ipecacuanha, which did great service in all cases but those where, from the patient's excessive weakness, it was contra-indicated. If the first dose of one scruple did not act, it was repeated, and in the interval a few grains of musk and camphor were given, which were found to assist the action of the ipecacuanha. These were continued till the temperature and pulse began to rise, and the skin to assume its natural colour. In the same circumstances the use of ice was followed with great relief. The patient received it in pieces as large as he could swallow, and his skin was rubbed with large pieces, and afterwards well dried with rough cloths, and the patient put in a warm bed. The first effect of the ice was to produce vomiting, which was soon followed by increase of the temperature, &c. Even in cases where the thirst, cramps, and oppression of breathing, were most violent, and the skin already cold and blue, with no pulse at the wrist, a reaction was produced by the employment of these means.

*Treatment of Third Form.*—In this form, the bleeding, ipecacuanha, and ice did more harm than good; most benefit was obtained from opium, by the mouth, and in injection with yolk of egg, camphor, musk, and especially the infusion of arnica.

Sinapisms was found of great advantage in every form; they were applied to the calves of the legs, and to the pit of the stomach, when the cramps and pain were severe. When the abdomen was tender to the touch, leeches and warm fomentations had a good effect. Vapour baths, hot sand, hot baths, were seldom productive of much good. In a few cases, a warm bath, in which caustic alkali had been dissolved, produced a reaction. Hot and stimulating frictions relieved the cramps in the extremities, but had no effect in raising the temperature.

Here then stand, on the one side, bleeding, emetics, ice and sinapisms, and, on the other, opium, brandy, camphor, oil of cajeput, sulphuric ether, hot frictions, baths, &c. These last were, at the commencement of the epidemic, almost exclusively employed. But in so far as the former were found to stop more effectually the course of the disease, to produce reaction, and to give an active character to those congestions which afterwards developed themselves, the use of the latter was almost unanimously abandoned by the physicians of Vienna, except in those cases of exhausting diarrhœa, or vomiting, described as the third form of cholera; for though they sometimes put a stop to the symptoms of the disease, and even roused the patient out of those desperate states of congestion or paralysis in one or two cases, yet their use was always followed by the sopor, and the worst forms of typhoid fever. Even in the third form they were given only in small doses, never in such quantity as was necessary to produce reaction in the first and second forms.

With the exception of the employment of ipecacuanha and ice to produce reaction, the physician must be guided by the general rules of practice, as there is perhaps no disease in which the treatment must be more varied according to the circumstances of the case.

"*Treatment of the Sequelæ of Cholera.*—When the bilious vomiting and hiccup were severe and long-continued, the best remedy was subnitrate of bismuth and ext. of hyosciamus; three grains of each were given every two hours till relief was obtained. Effervescing draughts. The sulphuric and nitric acids, in decoction of salep, or althea, were often equally useful. When vomiting and diarrhœa returned, during the convalescence, they were treated with success in the same way as the prodrome.

The patients were bled with the greatest advantage in those cases where the congestions were clear, and accompanied with strong reaction. In other cases, where the patients, on coming out of the cholera, fell into a state of stupor, or incomplete nervous fever, it was often difficult to determine if depletion or stimulants should be had recourse to. The one or the other was most successful, according to the individual circumstances of the case.

When the symptoms of encephalities came on, leeches to the temples, ice in bladders to the head, and sinapisms to the lower extremities, produced the best effects. In the few cases of inflammation of the lungs, tartar emetic, &c , were employed. When the belly was painful to the touch, leeches and sinapisms were employed; these were neccessary in almost every case.

When, after several days, typhoid symptoms showed themselves, such as delirium, black crust on the tongue, &c., no more good was derived from the continuance of the antiphlogistics—but, on the contrary, the most marked good effects, from small and frequent doses of musk and camphor, and opium, with infusions of arnica and valerina. When the patient remained weak, and had frequent returns of purging and inclination to vomit, the best effects were seen from an infusion of columba, gentian, cinchona, or other tonics, with a few drops of laudanum, and a diluted mineral acid. For a long time the least exposure to cold or wet, or the least excess in eating or drinking, brought on a return of the diarrhœa. The constipation, which often followed cholera, was best removed by a few doses of castor oil.

It is worthy of remark that the physicians of Vienna lay great stress on emetics and in sudorifics, and that *calomel* is not once mentioned as a remedy!

In a pamphlet of 30 pages Dr. John F. Henry, has embodied the results of his observation on the Epidemic of 1832, in this city. The whole work was a letter to his friend Professor Short, of Lexington.

Dr. Henry has launched out boldly upon the *ætiological* gulph, and discovered, as he believes, that the cause of Cholera is "*identical* with that of intermitting, remitting, and malignant fevers." As this hypothesis inculcates cleanliness—public and private, and the avoidance of night air, we think it not right to travel over the proofs which he has adduced in its support, for the purpose of showing their inadequacy. A corollary from this doctrine is set forth in a cheering prophecy, that Lexington, being little infected with misasmatic exhalations, would be but slightly visited, in the late visitation, however, she lost about 400, or a 15th part of her entire population, though more than half fled to the country, at the onset of the Epidemic. If it had depended on miasmata, from putrefying matters—animal and vegetable, how could this have happened?

We pass with pleasure to the second half of Dr. Henry's letter, which is devoted to the symptoms, prevention and treatment of the Epidemic.

"The disease in full developement was generally preceded by the premonitory diarrhœa. This I think was the most frequent mode of attack, but it was not always the precursor. In some very violent cases there was no diarrhœa during the whole progress of the attack. In one very remarkable case there was *no purging*, *or vomiting*, *or spasms.* He complained of a deadly intense pain in the epigastric region. His flesh was cold, that when you grasped his arm, you had that sort of chilling sensation which is produced by touching a dead person: the surface was bathed in a profuse clammy sweat, and his pulse was very feeble, and at times almost imperceptible. He remained in this state forty-eight hours, during which, there was no discharge of urine. After this protracted cold stage, reaction commenced; the pulse rose; the sweats became warm; the urinary secretion was re-established, and convalescence advanced slowly, though steadily. I saw three other cases in which the epidemic wore the livery of bilious cholic. There was obstinate constipation; but there were violent spasms of the intestinal tube, in one instance confined to the small intestines, and in the others involving the colon: but these cases could not be mistaken; they were but other modes in which the epidemic influence displayed its power: the same want of biliary and urinary secretion existed as in the ordinary mode of attack; and the remedies did not essentially differ from those employed in combating the common forms of disease.

At the same time, that these forms of cholera were desolating the city, I saw bilious fevers of every grade, from mild remitting to malignant. In others, on whom the influence was less operative, there were slight biliary derangements, indicated by nausea, or simple want of appetite, and by a torpid or sluggish condition of the bowels. Others had dysenteric symptoms with or without fever: women menstruated more freely, and at irregular times: but what was very remarkable, and different, I think, from what has been observed elsewere, I neither witnessed nor heard of any abortions during the whole period of the epidemic. Some cases that commenced as febrile attacks ran speedily into cholera; and, in my own observation, most generally into the stage of collapse; and others that commenced with all the symptoms of cholera, fell back into the milder forms of disease. There seemed to be a *scale* of danger, running as it were from simple biliary derangement to cholera in its worst form; indicated by the sudden supervention of diarrhœa, vomiting, prostration of strength, and pulselessness. This scale might be constituted in the following order; biliary derangement without fever—with fever of a mild type—of a more malignant grade—vertiginous state of the senses of seeing and hearing—numbness of the extremities—with costive bowels—with diarrhœa—with vomiting—with cramps—with strong pulse and warm skin:—with the preceding symptoms, but the pulse weak and vacillating, the heart having no power to send the blood to the extremties, or to the surface, which were cold, clammy, corrugated and livid or blue. Another scale might be formed, from observing the alvine discharges: where these contained the slightest admixture of bilious matter the danger was remote; and this danger was always in proportion to the thinness, on the one hand, and the approximation to the rice-water colour, on the other. All these discharges were bad—but worse in proportion as the colour became white."

The method he found most successful in the early or diarrhœal stage, was that which excited the functions of the skin and liver. The experience of our physicians generally, was to the same point. The following extract will show the means which he employed.

"In the treatment of diarrhœa, you should have the feet bathed in hot water, rendered stimulating by the addition of salt or mustard: you should then order the patient to bed; have hot bricks applied to the extremities, to the small of the back, and to the pit of the stomach. He should be covered warmly, and when free from nausea he should drink abundantly of warm tea of any of the sudorific plants; some prefer eupatorium, but I have found equal advantages from balm or sage, or table tea. In addition to these means, if the deficiency of temperature be great, sinapisms are ordered to the extremities, to the small of the back, and if nausea exists, to the epigastrium, and indeed to the whole abdomen: to these are sometimes added pulverised capsicum; or they are made more stimulating by being mixed up with spts. of turpentine, instead of hot water or vinegar. By all of these means, or such as you deem necessary in any particular case you restore the heat of the surface, and produce a copious perspiration, which you keep up until the disease is brought under the control of medicine; which consists for the most part of some preparation of *mercury* and of *opium*—ten grains of calomel, and one of opium, repeated every hour until the discharges from the bowels were arrested; and the calomel alone, in ten grain doses, until free discharges of a thick consistence, and of a green or black colour, were brought off, rarely failed to subdue this form of the disease. Sometimes the liver would speedily take on this mode of action; but at others, its secretions would pass through many changes before they reached the green consistent stool, which was ordinarily, the prelude to a healthy condition of this organ. Their transitions were very various; but the *lead* coloured stool generally semi-fluid, but sometimes firmer, and what I called the *charcoal stool*, rather thin, and having granules adhering to the surface of the vessel, resembling pulverized charcoal, were the most common. Any change from *white* to *green*, and from a condition of perfect *fluidity* to *consistence*, was favorable, and only required perseverance in the proper remedy to complete the cure. Sometimes, a single pill of calomel and opium was sufficient; but most frequently from sixty to one hundred grains of the former, and from two to six of the latter, were required. This, with such modifications as each physician preferred, has been the general practice here; and I have given into it; but a greater experience has convinced me, that a much less quantity of opium should be used; indeed, I am not sure that *any* is absolutely necessary, unless the spasms or cramps are present. Certain I am, that the congestion of the brain, so apt to occur after reaction takes place, is greatly aggravated by the opium.

If nausea or vomiting existed in the early stages, I gave an emetic of a saturated solution of common salt. Some used the mustard emetic, but I preferred the salt and water, unless the patient was greatly prostrated. The eupatorium was also given by many as an emetic; but either of the others are better in my opinion. I did not give the ipecacuanha at all. In one case I administered the antimonial wine, but I was not pleased with it, although the patient recovered. The advantages of an emetic, when nausea or vomiting existed, were very striking, (particularly of the salt emetic, concerning which my experience was considerable;) it threw off the contents of the stomach, and restored it to a quiet condition, in which it would for an hour or two, bear any medicine which the farther treatment of the disease rendered expedient. This was the golden period for the administration of calomel, or if preferred, calomel and opium. Sometimes I

have given half a dram or even a dram at a dose, and have been greatly pleased at the result. Indeed, I incline more and more to the opinion, that all you deem neccessary in the case should be given at *a single dose.* Some added to the calomel and opium a grain or two of capsicum, and a few grains of camphor; but I do not admire either addition, unless under circumstances, that would, in other diseases call for these remedies. I expected nothing more from the emetic, than what I have stated. I did not look to it as a means of rousing arterial action, and preparing the system for the use of the lancet; I confess I observed no such effects. On the subject of bleeding generally, my rule of practice was extremely simple; I was governed by the same indications which apply to this remedy, in other diseases. When the pulse is strong, no matter what its velocity, infinite benefit is derived from the lancet: when indistinct, feeble, and vacillating, it hurries on the fatal issue. I was always delighted, when I found a pulse that would justify the use of this instrument. But I bled to *reduce*, not to *increase* arterial action. For the removal of the *numbness*, free purgation with calomel was indispensable; without this, frictions did no good. In this condition I bled more freely than in any other, except when cramps were accompanied with *strong pulse* —a case of great apparent danger, but one which yields readily and beautifully to the lancet and calomel.

Vomiting, though a prominent, was generally the most easily subdued of all the symptoms of cholera. An emetic—a large dose of calomel, with or without opium—a sinapism to the epigastric region, or to the spine—quietude—withholding all manner of drinks, rarely failed."

Dr. Henry's experience has not inspired him with confidence in the efficacy of internal stimulants in the stage of collapse. On the contrary, he thinks them prejudicial. But he has nothing to offer as a substitute for them, and expressly says, you may do much to *avert* this state but nothing to *cure* it. We are strongly inclined to the same opinion. Patients undoubtly recover now and then, from this condition, and so they do from asphyxia, by drowning in water or carbonic acid gas, but these resuscitations are rather to be ascribed to nature than art. Those who survive the state of true collapse, do it, in all probability, from the inherent energy of the vital power, and would have lived, we suppose, if nothing had been done.

In conclusion, we may remark, that the pamphlet before us, though hastily and loosely written, is the production of a sensible and energetic practitioner, who saw much of the disease, and in his efforts to combat it, selected such means from our copious materia medica, as a man of talents and decision, would soon come to prefer to all others, which have yet been proposed.

---

Dr. Christopher Yates, of the city of New-York, in the 2d Edition of "Observations on the Epidemic," (August, 1832) finds two indications requiring to be fulfilled in its treatment:

"First. Remove as speedily as possible, the offensive matter from the stomach and intestines in the manner that nature indicates by her perhaps too feeble efforts, VOMITING and PURGING.

Secondly. Allay the spasmodic affection by anodynes."

This morbid secretion, in the opinion of Dr. Y. is the immediate cause of the vomiting and purging. Upon it he directs his measures, instead of looking to the irritation of the mucous membrane, by which the infiltration of an acid fluid into the bowels is occasioned. Let us hasten to his therapeutics.

"My remedy then is, to vomit or purge off the offending matter, be it what it may; and if pains or spasms continue to exercise the system *after* a free evacuation, especially by the bowels, give ten drops of laudanum every ten minutes until they cease; or at once give a full dose, of ether a teaspoonful, and laudanum fifty drops, mixed together in some liquid agreeable to the taste.

If the vomiting in this disease should be too incessant, and reject a full dose of an emetic, of which I prefer the tartrite of antimony (tartar emetic) before all others, I would, as I have been obliged in cases of yellow fever, dissolve twenty-four grains in eight ounces of water, and give a table spoonful *instantly* after each ejection from the stomach; in such case, eight or ten spoonsful have been taken, a small portion will have touched the coat of the stomach, when longer intermissions will soon follow, and eventually a complete emetic effect be produced. When once the first passages have been cleared, all danger from the disease is over; mucilaginous drinks, and occasional gentle laxatives finish the cure. This treatment is applicable to malignant cholera, and I shall rely on it for the treatment of the prevailing disease, which wants but one symptom to constitute it the Asiatic disease, and that is *cholera.* All the cases that I have seen in the hospital, which I have considered as ordinary disease, have in their commencement ejected bile. These will recover if they are not frightened into a worse stage, from their situation, or medicated into the disease. I would by no means have it understood that I would administer emetics in the forming state or first stages of the disease, unless accompanied with symptoms of nausea or vomiting. For premonitory symptoms I would and have now successfully used, a powder consisting of two parts of tartrite of potash (soluble tartar) and one of jalap—dose twenty grains every two hours till it operates freely; or, if more convenient, castor oil."

In a subsequent part of his pamphlet, our author remarks:

"There should in no instance be given any thing but mild cathartic medicines where there is neither nausea nor vomiting, nor a preternatural diminution of heat on the skin. In cases where the natural heat of the skin is either increased or diminished from its usual standard, accompanied with nausea or vomiting, I would have immediate recourse to an emetico-cathartic, composed of four grains of tartar-emetic and six grains of calomel.

Where spasms occur, I consider them merely symptomatic of the irritating effect of the secretion in the first passage of the intestines, and instead of soothing, paliating medicines, I would, by the first and most active means in my power, *remove* the morbid matter: and in case the symptomatic affections cease not with its expulsion, it is then in time to resort to palliative or antispasmodic remedies."

Dr. Yates asserts, that his practice has been eminently successful, and, in reference to the early and forming stages of the disease, we are prepared to credit his declaration. In the more advanced states it may, for aught we know, be as good as any other.

---

In the "Cholera Gazette," of Philadelphia, we find a notice of the various methods of treating the Epidemic in the Polish army. We shall transcribe but one of them.

"By Dr. Sturm, of the camp near Kamienka, warm water appears to have been the remedy chiefly employed. He gave the patients every fifteen or thirty minutes a glass of water as warm as it could be swallowed—he declares that after fourteen glasses at the furthest, had been taken, the disease was so far removed that the patient complained of a slight diarrhœa. The good effects of the warm water were, we are told, so promptly shown, that in two hours or even sooner, a cure was often effected, particularly when it was drank with sufficient freedom."

We see no objection to the idea that this simple practice may often be effective.

---

In speaking of Dr. Spencer's work, we took occasion to refer to the practice of Dr. Stevens. Among the treatises on Cholera which have reached us, is one by that gentleman, written expressly to recommend what is called the saline treatment. This method is based upon the acknowledged loss of the serum of the blood, with its saline ingredients. These, Dr. Stephens proposes to replace by throwing them into the stomach. Were they and the water in which they are dissolved, absorbed from the stomach, the practice would perhaps be beneficial; but where is the evidence of such absorption? We fear it does not take place. A surface which pours out so copiously as does that of the stomach and intestines, in most cases of Cholera, is not likely to absorb much of any kind of fluid. Moreover, as most of the saline solutions increase the serous discharges from the stomach and bowels, when given in other forms of disease, is it not possible, that they may do the same in Cholera, and thus prove injurious? This does not seem to us an extravagant supposition. It may be remarked still further, that Dr. S. proposes merely to supply a loss. He does not strike at the morbid condition of the solids, which drains the blood-vessels of their serum. But let our author, who is a man of intelligence, experience, and zeal, speak for himself as to the clinical effects of his practice.

"In the beginning of April, 1832, I received a visit from Mr. Pout, a medical gentleman in Albany street, who called to inform me that the Cholera had broken out in the prison of Cold-Bath Fields, and that he had been requested by Mr. Wakefield, the surgeon who had charge of the prison, to say that he would be glad to show me the cases; and from what he had heard of the saline treatment, he should be very willing to give it a trial—the more so, as he had now no longer any faith in the common remedies.

On the receipt of this message, I immediately went to the prison; and after some conversation with Mr. Wakefield on the subject, he not only agreed to adopt the saline treatment, but invited me to attend the cases along with him. He consented also that Mr. Crooke, a young medical gentleman who had lived with me for several years in the West Indies, should be allowed to remain constantly in the prison, to see that the medicines were faithfully administered, as well as to take notes of the cases.

The following is an outline of the practice which was pursued, not only in the prison, but every where else where I have had an opportunity of treating the disease.

First. The treatment was generally commenced with a Seidlitz powder, which was given with a view of lessening the gastric irritation, and partly for the purpose of removing the diseased secretions from the intestinal canal.

Secondly. When the stomach was irritable, (which it generally was,) a large sinapism was immediately applied to the epigastric region, and when the patients were cramped in the extremities, frictions were used with hot flannel. The pain produced by the spasms in the muscles was not only relieved by the frictions, but by this and the application of sinapisms to various parts of the body, the quantity of animal heat was increased, and this I need scarcely say, is an object of great importance in the treatment of Cholera.

Thirdly. A powder containing

Carbonate of soda, ℨss.

Muriate of soda, ℈j.

Chlorate of potass, grs. vij.

was dissolved in half a tumbler of water, and given soon after the seidlitz. In severe cases, the above powder was administered every half hour. In those that were less severe, it was used every hour, and in some malignant cases it was given every fifteen minutes. In short, it was given more or less frequently according to the circumstances of the case, and continued until the circulation was fairly restored; it was then given at longer intervals, and when the reaction was completely established, it was left off by degrees.

Fourthly. When the stomach was irritable, the use of the above powder was occasionally suspended, and common effervescing mixtures, or small doses of the common soda powders, with an excess of the carbonates were frequently used, until the irritation was lessened, and then the carbonate of soda, with larger doses of the chlorate of potass were generally given without the addition of the muriate of soda, and frequently in such cases the chlorate of potass was given by itself, in doses containing ten grains each.

Fifthly. A solution of muriate of soda was also thrown up into the intestines, at as high a temperature as the patients could well bear this saline fluid.

Sixthly. In two very severe cases which occurred out of the prison, the patients were put into a hot saline bath with evident advantage. It is well known, that a hot saline fluid is a better conductor of heat than fresh water at the same temperature; but, independent of this, a part of the saline ingredients may be absorbed from the skin, and the patients may also be benefitted by respiring the hot saline vapor. It is but fair to state, however, that this means, which was evidently beneficial in the cases in which it was tried, was proposed by Mr. Marsden, one of the surgeons to the Free Hospital in Greville-street.

Seventhly. Soltzer water was allowed *ad libitum*, when the patients expressed a desire for something to drink. A strong infusion of green tea was also occasionally used, in severe cases, apparently with advantage.

Eighthly. It was considered essentially necessary to keep a large fire, both night and day in every room where there was a patient with Cholera. It is now well known, that in by far the majority of cases, the collapse commences betwixt two o'clock in the morning and six A. M., or in other words, at the period of the twenty-four hours when the atmosphere is coldest: from which it appears that external cold acts as an exciting cause to the state of asphyxia. But independent of this, we have seen that the degree of force, with which oxygen can remove carbonic acid, through the medium of a membrane, depends, in a great degree, on the temperature of the two fluids. Now, when the temperature of the blood is so very low, as it is during the state of collapse, and if the air which the patients then breathe be also cold, the small quantity of carbonic acid which exist in the black venous blood, will not be attracted by the cold air, and consequently this of itself may be one cause of the sudden death.

Ninthly. It is necessary to be very careful not to dismiss the patients as cured until they have been, at least, several days completely out of danger. Two of the cases which proved fatal in the prison, at Cold-Bath Fields, were lost, from our not having been at that time sufficiently aware of the importance of this.

Tenthly. The patients ought not to be allowed to use one particle of solid or indigestible food, for at least five days after they have recovered from the state of collapse. We nearly lost more cases than one, from the too early use of solid indigestible food; and one woman, a nurse in the London Free Hospital in Greville street, actually died from this cause, after having been considered as completely out of danger from a most violent attack of Cholera accompanied with collapse.

Eleventhly. Those who put their patients under the saline treatment, ought to trust almost entirely to this; for if they use calomel, brandy, or other destructive agents at the same time, they will do little good; but above all, not one particle of opium ought to be given internally; for, from what I have seen, I consider this to be as fatal in Cholera, as it is in the last stage of either the African typhus or the seasoning fever of the West Indies. Where the stomach, however, is extremely irritable, about twenty-five or thirty drops of laudanum, diffused in a little tepid water, may be injected with a small syringe, into the rectum, not only with impunity but considerable advantage.

When the stomach is very irritable, small quantities of milk with carbonate of soda may be given occasionally; and when we use the saline powders in such cases, they ought to be dissolved in a very small portion of water.

When the case is exceedingly malignant, or where we are called in late in the disease, and find the patient already in collapse, we ought then to have recourse to the most active measures. An ounce of the muriate of soda, with half a drachm of the muriate or the chlorate of potass, should be given immediately in cold water and repeated, if necessary, every half hour, until the patient has taken about three doses of this strong solution. Should reaction be brought on by this, it may then be kept up by the common saline powders; but should this experiment fail, we may then, as a last resource, give the patient another chance for his life, by injecting a saline fluid into the veins.

The ejections, and every other source of impurity, ought to be immediately removed from the room where the patients are; and the infected wards should be fumigated at least twice a day with gunpowder, and every particle of suspicious clothing, bedding, &c., should be boiled, for at least half an hour, in a strong solution of common soda. Those who are recovering from the disease are liable to a relapse, and such cases are generally fatal; but from what I have seen, my belief is, that those who have completely recovered after having had the Cholera once, have an immunity from any future attack of this disease.

The above is an outline of the treatment and means which were used: the following is, I believe, a fair statement of the outline of the result:—

The three first cases which occurred in the prison were treated by Mr. Wakefield in the common way—with opium, brandy, the hot-air bath, &c.; but they all died after a very short illness. Almost immediately after this, another case was treated in a similar way by another practitioner, who had been sent for to the prison during the night, in consequence of Mr. Wakefield being unwell at the time. This gentleman was not then aware that any new practice had been adopted in the prison. He treated the patient *secundum artem*, with brandy, opium, and chalk; but the result was, that this patient was past all hopes of recovery before either Mr. Wakefield or myself saw him in the morning;—consequently the four cases that were treated in the prison in that way, there were four deaths and not one recovery under the common practice.

It may be proper to state, that previous to the beginning of April there were no bowel complaints in the prison, and the whole of the prisoners were then as healthy as they generally are at that season of the year. The first case that was reported to the Board of Health occurred on the 5th of April;—the saline treatment was commenced on the 8th. There were in all at that period about one thousand three hundred souls in the prison; and from the 8th of April to the cessation of the first epidemic, there were at least one hundred individuals who were evidently more or less, under the influence of the poison.

In about fifty of the above cases, the patients were attacked with a bowel complaint, and most of them had, more or less, irritation at the stomach. The fluids that were ejected, were generally deficient in bile, and the bowel complaint was attended with the following peculiarities:—

First. The inclination to go to the night-chair came on more suddenly than it generally does in cases of common diarrhœa.

Secondly. The ejections were less bilious than in common diarrhœa; and opium, chalk, astringents, &c., which are generally useful in cases of common bowel complaints, were of no use in checking the diarrhœa, which occurs when the patients are under the influence of Cholera poison. These remedies were chiefly used in cases which occurred out of the prison; but they evidently had no effect in checking the specific ejections which are produced by the Cholera poison; and this I presume was the cause of the diarrhœa which occurred in the fifty cases in the prison to which I refer. The whole of these were immediately put under the saline treatment, and this appeared to give an immediate check to the disease; and I believe it was owing to the saline remedies, as well as to the circumstance of their being constantly kept in a warm room, and well taken care of, that not one of these cases ended in collapse: consequently, though they were constantly breathing in an atmosphere completely impregnated with the poison, yet no one of them was lost.

There were also about thirty-one similar cases, in which the above symptoms were still more distinctly marked, and in many of them the bowel complaint was more or less accompanied with cramps. These were all treated in the same way with the non-purgative salts, and in three, four, or five days, every one of them were sent from the observation ward, as we believed at the time, completely out of danger. I am sorry, however, to be obliged to add, that two of these cases which had been unfortunately dismissed too soon, and sent back as cured to the cold wards of the prison, were attacked with collapse during the night, and before they could again be put under the saline treatment, their stomachs were so irritable that they could scarcely retain even a tea-spoonful of water; and both these cases proved fatal in a very short period from the commencement of the collapse.

In addition to the eighty-one individuals already referred to, we had about nineteen cases in the prison, where the patients were either attacked with the disease, and got into a state of asphyxia in the cold wards of the prison during the night, or where the stomach was so irritable in the first stage that it could not retain the stronger salts. In almost every one of those cases the disease assumed a most malignant character. These were all treated with the energetic non-purgative saline remedies; and in the nineteen malignant cases to which I now refer, we had eighteen recoveries and only one death: consequently, the total number of patients, who were all evidently under the influence of the Cholera poison, was about one hundred, yet in those cases where we trusted almost entirely to the saline practice, we had only three deaths, and ninety-seven recoveries.

The saline treatment has now been used, in that part of London, in about two hundred and twenty-six cases of Cholera. Out of this number there have been about twenty deaths, and upwards of two hundred recoveries. It is true, however, that many of these were not cases of collapse; for this, where we saw them early, was generally prevented by the immediate use of the saline treatment; but from what I have seen, my conviction is that, if these cases had been treated with the common remedies, the one-half of them would have been lost. Or, when we compare the result, even in the most malignant cases, with the average mortality not only in London, but in other places, it will be found that the balance is greatly in favor of the saline treatment."

Let us recur to the supposed *modus operandi* of this practice, Dr. S. has, as he informs us, made experiments which have satisfied him, that the red color and stimulating qualities of the blood, depend, either directly or indirectly, on its saline ingredients. When these are wanting, the oxygenation and decarbonization of that fluid cannot go on. By supplying them the great function of œration is restored. The altered condition of the blood, in cholera, yellow fever, and other malignant diseases, he regards as primary, the affection of the solids as secondary. The ærial poisons which engender these maladies, he supposes to be absorbed through the lungs, when they deteriorate the constitution of the blood, and promote the excretion of the serum with its saline ingredients, or effect their decomposition and develope muriatic acid in the circulation, which is often, he affirms, discharged upon the mucous membrane of the stomach and bowels, in the diseases to which we have referred.

Dr. Stevens' labors seem not to have found favor with the Medico-Chirurgical Review; nor does his practice appear to have been generally adopted in the United States. Our own trials have been too limited, and too much mixed up with other measures, to enable us to speak with the least authority. We hope our readers will think the method worthy of being submitted to the test of a candid experience.

The 22d and 23d Nos. of the American Journal of the Medical and Physical Sciences, contain two long and elaborate papers on the Epidemic, by Dr. Jackson of the University of Pennsylvania. They are characterized by the minuteness of observation, acuteness of thought, and copiousness of technical language, which distinguish that able and eminent man.—Without attempting to follow him through his course of elaborate pathological speculation, we shall endeavor, in pursuance of the plan of this review, to present his *methodus medendi.*

He divides the disease into five stages.—1, Slight cholerine or diarrhœa; 2, confirmed irritation, attended with vomiting, purging and cramps; 3, incipient concentration, or early collapse; 4, confirmed collapse; 5, reaction or consecutive fever.

We shall transcribe his summaries of the treatment, which he conceives to be the best adapted to these different stages. The extract which follows, relates to the two first stages.

"The *indications of cure* founded on the foregoing principles, are the following:—

1st. To adapt the aliment as to quality and quantity to the state of the digestive forces; or to suspend entirely, or greatly to diminish the exercise of the digestive functions.

2d. To allay the sanguine and nervous irritation existing in the whole alimentary canal.

3d. To bring on diffusive excitement, or the irradiation of the organic circulation, counteracting and preventing its concentration towards the alimentary mucous tissue.

4th. To accomplish a more healthy state of action, of the secretions, and regular functional performance of the bowels.

The practical, remedial and therapeutic means, for the accomplishing of the preceding indications, are various and diversified. They are to be employed and adapted according to the particular case characterized by its distinguishing symptoms.

For the attainment of the first indication, the diet of the patient should be made to consist, in the first period of the disease, of boiled black tea, with cream, and stale wheat bread, or soda biscuits, for breakfast and supper; while rice, grits, or homminy, should constitute chiefly the dinner. Weak chicken or mutton broth, or water, is frequently most soothing and grateful to the stomach. Toast and water, barley or rice water, form the most appropriate drink. In the second period, or confirmed irritation, the diet must be absolute, or consist solely of chicken water, rice or barley ptisan.

The second and third indications are accomplished by nearly similar means; for the attainment of the one almost necessarily induces the other. These means are, 1st, immersing the feet in a warm stimulant foot-bath for fifteen or twenty minutes: the bath may be sinapised or made of a weak alkaline ley or solution of salt. The patient is then to be placed in a bed well covered, with warm applications to the feet, as bottles of warm water, warm bricks, irons, or bags of heated oats, &c. If there be much uneasiness in the abdomen, warm epithems are to be applied, and renewed as they become cool. Hops steeped in warm water or vinegar and water, and subsequently expressed nearly dry, I found very convenient for this purpose, Dry heat is to be preferred in some instances, and then a stomach warmer, or heated oats, hops, or salt contained in bags, may be resorted to for this object. In the lighter forms of the first period, or the preliminary and premonitory stages of the disease, advancing into the confirmed affection, the above treatment very generally suffices to dispel the symptoms, and arrest its progress in the course of one or two days. The observance of a proper regimen is all that is, then, requisite to protect against a relapse. This I found was the greatest difficulty in the management of patients. The restoration to a state of comfortable feeling was considered as a license for indulgence, but as the specific predisposing cause is in constant operation during the epidemic period, the necessity for attention to the prophylactic or preventive regimen still continues. It is even more imperative, as the attack already suffered, leaves the alimentary surface more irritable, more sensitive to impressions, and of consequence, more disposed to pertubating actions from light exciting causes.

2d. The administration of diffusive excitants in small doses, in order to ensure their introduction into the economy, and the general or diffused excitement through the organism. One of the most frequently employed of these, was the alcoholic solution of camphor. The partizans of Hahnemann and the homœopathic doctrine, extol camphor as a specific; but, from my own experience of its operation, I am satisfied that its action is diffused excitement. Taken in small doses, I have found a sentiment of warmth and glow to be developed on the surface, succeeded by a gentle, warm transpiration. This is the precise reverse action of that to which a constant tendency exists in the first and second periods of the disease, and which is counteracted by the action of camphor. The Hahnemannists condemn its association with opium in any shape. My own observation does not confirm the correctness of this interdict. On the contrary, I found great advantage from the combination; and the cases in which the camphor proved most useful, were the light forms of the first period, with nervous pains of the abdomen. The dose in which it is administered, is from two to five drops, mixed with cold, sweetened water, and given every fifteen minutes, half hour or hour, according to the urgency of the symptoms. The premonitory symptoms were, in immense numbers of cases controlled by this remedy, and in the incipient stages, I have seen the most decided effects when the symptoms were principally of a nervous character. Other mild diffusible stimuli were however of equal utility, which is an evidence that in camphor there is nothing of a specific mode of action. Aqua ammoniæ in the dose of two drops, combined with five or ten drops of tinct. opii, and given in sweetened water, answered quite as well. The following prescription I frequently used, and found it to succeed equally with the camphor in the same class of cases:—

℞. Tinct. lavend. comp. tinct. camphoræ, āā. ℥iv.; liq. Hoff. anod., tinct. thebaic, āā. ℥u. M. ft. mit.

From ten to twenty drops of the above were administered at appropriate intervals.

In the cases in which the nervous symptoms were less decided, I generally directed the following prescription.

℞. Massæ hydrargyri, gr. viii.; ext. opii. aq. pulv. ipecac., āā. gr. ii. M. Divid. in pill. iv.

One of these pills was given every two, four, or six hours, according to the symptoms.

When the inflammatory symptoms were not very acute, the symptoms frequently became mitigated by the preceding remedies. But whenever the vomiting and purging continued, accompanied with severe pain in the abdomen, then I had recourse to sanguine depletion, either general or local; or both, according to circumstances. General depletion was practised in the limited number of cases, in which the pulse was full and excited; but in the others, leeches or cups to the abdomen were found the more effectual and safer proceeding. It is surprising to witness how prompt often is the alleviation and abatement of the symptoms, following the application of from thirty to sixty leeches to the epigastrium and the illiac regions. In no instance did I find it necessary in the patients I treated in the first stages, to repeat the local depletion. One application was sufficient to procure a favorable result. In several instances general depletion made but little impression, while a very prompt relief ensued upon local depletion.

General blood-letting is but partially applicable to the cases of cholera. It should be restricted to those only where the constitution is vigorous, and tbe patient has not been enfeebled by age, previous diseases, or dissipated living; and where the forces of the general circulation do not exhibit a manifest tendency to failure. In those cases in which it proves an available and salutary remedy, its operation is the reduction of the internal irritations, and the inviting to a revulsive movement, co-operating in this mode with, and facilitating the diffusive or centrifugal action of the means put into operation for this object. This importaat movement, the *great recuperative action* of the organism in the *natural cure* of diseases, is based upon the vital forces of each organ, the susceptibility and capacity of each organ to assume and maintain vital reaction. Now, this state depends on the activity of the organic circulation in each organ, and its supplies of the vital sanguine fluid, the great energizer of vital reaction.

If, therefore, this be abstracted below the sum necessary to maintain the vital reaction of each organ in the degree essential to its functions, a state of asthenia, sedation, or exhaustion, is the necessary consequence, and diffusion of irritation, irradiation, reaction, and a centrifugal movement, tending to the equalization of the organic and general circulations and the forces of life, become nearly or quite impossible. Whenever the signs of concentration are rapidly approaching, or the state of collapse, so named, is imminent, general depletion is then a most equivocal, nay, a hazardous remedy. Should it fail to remove or greatly to lessen the centrifugal force of the internal irritation, and produce a revulsive operation, its effects must be most disastrous. Its direct action being the abstraction of blood from the general circulation, the force of this, the energy of the heart's contractions, the supplies of sanguine vital fluid sent into the organs for the sustentation of their vital reaction, all are diminished, and of necessity the whole organism is prostrated. The sedation resulting from general depletion, cannot, from its mode of action, be directed so as to influence those organs exclusively whose vital reaction is in excess. All are influenced alike, and the revulsion or diffusive excitation, which is merely favoured by general depletion, is dependent on other causes, which we neither can absolutely command, nor whose presence even can we know by any positive signs. To resort to this remedy, therefore, when the failure of the forces of the general circulation evidences the exhaustion already iuduced in the sum of the sanguine vital fluid by the concentrative energies of the internal irritations, is to throw ourselves on the chapter of accidents, with this serious disadvantage, that the loss of our hazard is irretrievable ruin, must almost inevitably prove fatal.

Local or organic depletion offers none of the objections or difficulties attendant on general depletion. Its mode of action is totally distinct, and it is applicable to numerous cases where general depletion is entirely inadmissible. In local depletion the blood is not abstracted from the general circulation directly, and its influence is not that of a direct debilitating remedy immediately producing an exhausting, debilitating action on the whole organism. Its operation is restricted to the organic, or as it is termed sometimes, capillary circulation, and is attended with a local irritation or excitation, resulting from the means by which it is accomplished. The common universal result of local irritation or excitation is thus produced—an afflux towards the part irritated or excited—but the escape of the blood, preventing the formation of a congestion, which arrests this movement when a simple rubefacient is employed, and limits its influence, renders it continuous when caused by local depletion, extends it into a wide, expansive area, and often causes a decided and marked affluxive movement through the whole organic circulation. By the establishment of this movement, a direction is given to the organic circulation towards the point where the local depletion is made and the blood is escaping; towards this point does the sanguine fluid tend, and diminishes consequently in those the most opposite, and hence arises the peculiar and admirable effects to be obtained by the employment of these means—effects more frequently applicable in the treatment of diseases, and of more decided and beneficial agency than most other therapeutic remedies at our command. It is a remarkable fact, that the debilitating or sedative action of local or organic depletion is at the points the antipodes of that where the depletion is made. The application of the means used for this purpose is not a matter of indifference. The position selected for the application in most instances, there are exceptions under certain circumstances, should be directly opposed to that intended to be influenced. Were this the place, and the digression would not be too long, I could cite numerous facts in support of the axiom. A very little experience with these means is sufficient to give the demonstration even to casual observers of its accuracy. Local depletion from the thorax, it is known to all, will not give relief in the inflammations of the abdominal viscera, nor when made from the abdomen, prove beneficial in the inflammation of the thoracic organs. Did local or organic depletion accomplish its effects merely by the abstraction of blood, it would be indifferent whence the blood was taken, so that the amount was the same; but the contrary is too well known to be contested.

In what manner local depletion causes this affluxive movement in the organic circulation, and expends its influence on tissues different and remote from that whence it is made, can no more be explained than can the same operations be explained as resulting, though in a lesser degree, from the action of rubefacients, vesicatories, cauteries, and other revellent measures. These operations depend on the forces and laws of the organic, vital and molecular circulation or movement of the sanguine fluid in the tissues. The mechanical or general circulation—the movement of the sanguine fluid in masses or columns to and from the respiratory surfaces, and to and from the nutritive surfaces or organic tissues—accomplished by physical forces and on physical laws, has been well investigated, and is almost, though not completely, elucidated. But as respects the organic textular circulation, our information is very limited. We know nothing of the forces producing and maintaining, and but partially the laws that govern it. It is dependent on the same forces, and governed by the same laws, that are the causes and regulators of vital reaction, or the phenomena of vitality. As the mode of action of these forces is unknown, inscrutable, it is not possible to assign the mode in which this great vital phenomenon, so deeply concerned in the pathological condition, and of so great a moment in therapeutic operations, is accomplished. We must therefore be contented with the facts revealed to us by observation, and attempt the establishment of those general principles or laws—the coordination of the facts in the order of their dependence, which are obtained by a well-regulated experience.

In addition to the foregoing means, I prescribed, when the stomach was much distressed with nausea and vomiting, the effervescing draught of Riverius, to which was added the black drop. Frequently

I made the solution of the bi-carbon. potassæ in the aqua camphoratæ, according to the following prescription:—

℞. Bi-carbon. potossæ, ʒiv.; acetat. opii, gtt. xv.; aquæ camphoratæ, ℥iv. M. ft. solut.

Half an ounce, mixed with an equal quantity of lemon juice, was given in the act of effervescing. The dose was repeated every hour or half hour. This draught was often very grateful.

To allay the thirst, and at the same time to abate the gastric irritation, iced water in small quantities, or iced gum water, very slightly acidulated with lemon juice, and sweetened, was allowed for drink. The iced carbonated or Seltzer water was also a general beverage, and highly grateful to the stomach. In many instances ice itself was given in very small pieces. One of the patients I attended early in the epidemic attacked with the disease, was so delighted with the relief he experienced from iced drinks and from ice, that when I subsequently saw him, he would cry out "give them ice."

By allaying the intense sensation of thirst, which often amounts to agony, one of the sources of nervous disturbance and irritation is cut off. Cold also acts as a direct sedative on the gastric mucous membrane, and the decided relief obtained by its employment, while it establishes the propriety of the treatment, proves clearly the highly irritated state of that organ, and the kind of agency resulting from cold drinks. The diminution of the irritation of the gastro-intestinal surfaces, by weakening the centripetal action, is the most efficient step towards establishing a diffusive movement, in promoting irradiation or reaction, and rendering more efficacious the operation of the external revellents, the cutaneous reaction caused by sinapisms, frictions, heat, leeches and cupping, or the force producing a centrifugal movement.

4. The last indication in the treatment of the first and second periods, or of cholerine, in the cases in which it arises, and in my practice they were very few, is attained by the administration of mild laxatives or eccoproctic remedies. The treatment already indicated in the cases under my charge, was generally adequate to the entire protection of the individual from the invasion of confirmed cholera, and to the perfect restoration to a healthy condition of the alimentary canal. In two of fifty-five patients, a costive disposition ensued, which continued for several weeks; but finally disappeared by the combined employment of appropriate regimen and laxatives. In one, an alkaline infusion of rhubarb was employed; in the other Saratoga water. In two, more uncomfortable feelings, and occasional tormina, continued to recur at intervals. This was removed in one by fifteen grains of rhubarb; in the other by small doses of calomel and opium, with castor oil in the dose of one drachm. These were the only cases in which I found it necessary to prescribe in order to bring the bowels into a natural condition after the cholerine symptoms had been removed. They were the only instances also in which I employed any purgative or evacuant medicines."

His principal remedies for the first stages of collapse, were the effervescing draught, camphorated water, mucilages, ice, laudanum, half grain doses of calomel, astringent injections, and sinapisms. His treatment of the state of confirmed collapse, we shall give in his own words:

" *Treatment.*—Adopting the above pathological views of the proximate causes of the fourth period, or state of collapse, the indications of treatment become well defined. They are, 1st, to arrest the inordinate evacuations emptying the vessels, enfeebling the heart, and exhausting the circulation through the stomach, bowels, and skin. 2d. To restore the wasted fluids by giving to the patients constantly drinks of various kinds, and administering aqueous enema intended to remain in the bowels. 3d. To continue the treatment calculated for the first periods of the disease, which is to allay the extensive irritation of the alimentary mucous membrane. 4th. To quiet the irritation of the nervous system causing spasms and cramps: and 5th. To produce as early as possible diffusion or re-action.

The first of these indications is fulfilled in different manners. The safest in the cases of the temperate and robust, is the direct antiphlogistic treatment. This consists in allaying the vomiting by the effervescing draught; iced carbonated water; small pieces of ice constantly held in the mouth: leeches or cups to the epigastrium and abdomen. General depletion, when collapse is threatened is highly dangerous. It is admissible only in the first periods and in robust individuals, especially of the sanguine temperament. When the circulation is already enfeebled and failing, the abstraction of blood from the emptied vessels is a measure calculated to insure exhaustion, the proximate cause of the collapse. Subsequent to the employment of these, or at the same time, lime water and milk may be administered every hour. When spasms exist, opiates in small doses, and camphorated water are demanded, and are often useful. Calomel in the dose of gr. ss. to gr. i. with acetat. plumbi, gr. i. given every hour or half hour will succeed in some instances.

The same end is attained by an opposite method, and is best adapted to those who are threatened with rapid exhaustion. It is not as safe in the more robust individuals, and must not be persisted in for too long a period. This method consists in direct or immediate revulsion, converting the secretory irritation of the alimentary mucous membrane into a higher grade of action or inflammatory irritation, and thus arresting the evacuations. In this manner do the mustard and brine emetics operate, as also ipecacuanha, and some of the stimulant and irritating cathartics employed. Calomel in large doses, by changing the mode of irritation, will accomplish often the same end. When the evacuations have been controlled by the remedies of this character, a new danger ensues. The patient has to encounter the hazards of a gastritis and enteritis, with a febrile re-action having those local affections for its basis, and attended often with secondary sympathetic determinations to other organs of great intensity. The excitant, direct revulsive treatment must be immediately abandoned when the signs of these appear, and the antiphlogistic substituted in an appropriate manner.

The cutaneous transpiration proves exceedingly exhausting to the patient, and is of itself sufficient often to bring on collapse. It should never be excited by surrounding the patient in blankets, or enveloping him in warm vapor. Prior to our own experience of the disease in this city, and relying on European statements, the sanitary committee, acting on the advice of the Consulting Medical Board, made most ample provision of various heating apparatus, steam baths, stomach warmers, or other similar means. All the bedsteads of the hospitals were adapted for the use of steam, and in the management of the first cases these means were assiduously employed. No patient under this treatment survived over from four to six hours, although some had strength sufficient to walk into the hospital. Heat was most distressing to the patients, who resisted its application, and protested often vehemently against its employment. It must be confessed, that the prejudices and excitement of the public against the hospitals in the commencement of the epidemic were not without foundation. This method was soon abandoned. The patient succumbs the more rapidly in proportion to the profuseness of the transpiration. It is best checked by cool air, frictions made with cloths dipped in iced water, or with ice itself, followed up immediately with dry frictions. Frictions with actively irritating liniments, with dry chalk, and similar measures, were found often effectual in arresting the cutaneous discharge. The evacuations from the bowels are best restrained by small doses of opiates, or the same given in enema, either of simple water, mucillage, or saline solution. In some cases astringents are useful. I prefer of this class the rhatania.

The second indication is obtained by administering freely of drinks and liquids to the patient. While the stomach is very irritable, and the thirst intense, ice, iced water, iced Seltzer water, or carbonated water are the most grateful to the patient, and the most frequently successful means. In persons who have been addicted to the excessive use of ardent spirits, some stimulus is often required, as small portions of brandy, wine, or carbon. ammoniæ, aq. ammonia, or others of this class. The weak animal broths, as chicken, mutton, or veal water, were found very useful. Gum, rice, and barley water were advantageous, as allaying irritation, affording light nutriment, and replenishing the circulation.

The third indication is fulfilled by the same means recommended for the first and second. They allay irritation, and it is by that operation they arrest the vomiting and the profuse, exhausting secretions and evacuations. When the direct revulsive treatment was followed with a view to suppress the evacuations, as soon as that object is accomplished, the remedies must be cautiously employed, or entirely withdrawn; as then, they oppose the obtaining of this indication. Small doses of opium and calomel, or opium, calomel and ipecacuanha are adapted to this end—together with opiate enema.

The last indication can never be obtained until the evacuations have either entirely ceased, or are very considerably diminished. At least, such is the result of my observations. While they continue, and the circulation, general and organic, is reduced to a low ebb, and the vital forces in all the organs nearly extinct, diffusion or irradiation of excitement cannot be accomplished. For this purpose, mobility of the organic fluids, (the fluids of the capillaries,) is indispensable.

The most effectual means are cutaneous revellents, and the safest direction to give the excitement is to the skin. Frictions, dry, or made with irritating liniment, sinapisms and blisters to the obdomen and extremities, by exciting the cutaneous tissues, a'e the most efficient means of rousing the dormant forces, and of inviting the excitement from the internal surfaces to the exterior. The diffusible stimulants may be brought into action with this intention in many cases. The best, most certain, and safest mode of employing them, is in minute doses frequently reiterated. In this manner their absorption and diffused action is procured, while their local excitation is to a great extent obviated.

Vigilance is required in watching the re-awakening of the forces, and the production of re-action. It is not uncommon for it to occur in a single organ with great rapidity, and to occasion a fa'al result. Apoplectic symptoms in this manner are often the immediate cause of death. In one case to which I was invited in consultation, a suffocating catarrh in one lung, with pneumonia in the other, were developed in the re-action, and destroyed the patient in a few hours, from the termination of the collapse.

The fourth period, or confirmed concentration and collapse, when completely established, must necessarily prove fatal in the great majority of cases. It is a moribund state. No method of treatment can be more than partially—very partially successful. The great object should be to prevent the disease from arriving at this period. The best system of treatment is that under which, in any given number of cases, the fewest reach this stage. The mortality of the disease is then inconsiderable. When collapse has been induced, the results of different methods will not vary to any great extent. No one can boast of more than few and scattered triumphs. The mass will and must perish. The vital lesions are too considerable, and vital functions too profoundly disabled to be restored with certainty by any human means.

It is an argument used by some, that because the patient is in a most critical and dangerous position, that, therefore, violent treatment and powerful remedies must be resorted to. This arises from the employment of figurative language and the personification of disease. The disease is a giant, and pigmy remedies cannot overthrow it. Such or similar expressions are employed. But the disease is nothing separate, nothing distinct from the organs. It is a modification, and effects arising from the modification of the organs. Remedies cannot influence disease independent of the structure. They are modifiers of the vital activity and movements of the tissues. When the vital forces are prostrated to the exceedingly low ebb, they are seen to be in the period of collapse, the vital movements in the tissues nearly extinct, and the vital functions reduced to their lowest state. is there not a greater probability that violent—energetic treatment so called—and excessively perturbating remedies, will extinguish the feeble remains of life, rather than recall them to their wonted activity? This has been my impression, and such I believe the evidence afforded by my opportunities of observation."

Dr. Jackson's observations were made in a Cholera Hospital, of which he was physician-in-chief. It was open 34 days. The number of patients treated was 69, of whom 20 died.

The method pursued by Dr. J. is negatively characterized, by the comparative omission of calomel, internal stimulants and external heat.

---

We are indebted to the Gazettes for the experience and speculations of Professor Dudley, on the nature and treatment of Cholera. We have already spoken of its prevalence and great mortality in Lexington, Kentucky. During the visitation, that distinguished surgeon expressed himself as follows:

"While there is considerable variety of opinion about the remedies to be used, there can be no difference among medical men, about the necessity of correcting the morbid watery discharges, and of establishing thick or consistent evacuations from the bowels of every patient, the subject of cholera. Every physician and nurse concurs in sentiment, that upon the accomplishment of this object, depends the immediate safety, and the ultimate recovery of the sick; while it is admitted by all, that death must supervene where this is not effected."

When this is accomplished, the disease is, of course, already at an end. The condition of the digestive organs here spoken of, is a condition of health.

"The suspension of the healthy action of the stomach, liver and kidneys, in cholera, is familiarly known to every observer: nor is the pale, shrunken and cold state of the surface, with a consequent accumulation of blood in the internal organs less satisfactorily understood. With these facts before him, recorded in the books, and made manifest in the appearance of the cholera patient, the practitioner is required to trace out the general principles by which he is to be governed in the administration of medicine."

Dr. D. recognizes in Cholera, the subversion of other functions than those of the stomach and bowels, but keeping the latter constantly in view, he proceeds to form his indications from their condition.

"Whatever the cause of the epidemic may be, the principal force of it is spent upon the digestive organs, and especially the stomach and bowels; while it is upon the same organs that medical impression is made with a view to cure. So far then as the cause of cholera may be conceived to be located in the stomach, emetics are suggested to the mind with a view to its removal, just as a person who had swallowed a dose of arsenic would expect relief from the same remedies."

We are scarcely prepared to go with our author in the views set forth in this paragraph. The Epidemic poison would seem to be one of the greatest subtility. It has certainly eluded *all* observation. Such a poison *may*, we admit, be swallowed, but where is the proof, whence the probability? The deglutition of an ethereal fluid, or of *such* a gas, as we may presume to occasion Cholera, would, we suspect, be rather a difficult function, and then, what is to detain in the stomach, a material of such great specific levity? Do not the gases generated there, escape perpetually by eructation? The idea of administering emetics for the purpose of expelling this rarefied poison, as we would expel a dose of arsenic, although entertained by Darwin, does not strike us very plausibly. Nor do we consider it necessary to employ an emetic for the purpose of arresting an attack of Cholera. Such an arrest may be very certainly effected, by means entirely different, in their *modus operandi*. A pediluvium, a warm bed, herb teas and a dose of laudanum, or Dover's powder, in the beginning of the malady, will commonly allay the gastric irritation, and avert the more formidable symptoms although they evacuate no poison, either solid, liquid, gaseous, or ethereal.

"I have long been in the habit of using active emetics successfully, in the convulsive forms of *hysteria*, proceeding from derangement of the digestive organs; and in cholera of the spasmodic form, I have not been less pleased with their effects; since they invariably mitigate, and generally subdue the disposition to cramps altogether. By the mechanical agitation of the internal organs from free vomiting, or high efforts to vomit, the blood is driven to the surface of the body, the coldness of the skin removed, the internal organs relieved in part of congestion, and the vital energies restored, where any power of reaction is left. Where reaction cannot be effected by emetics, the system is insusceptible of any impression. This remark is based upon the fact, that I have seen more cases terminate in death, without any manifestations of reaction, where no emetics had been used, than where they had been given with a liberal hand.

Again; emetics possess the power of suspending watery secretions into the bowels, when administered so as to excite free vomiting. But above all, I conceive them valuable, as possessing the faculty of arousing the stomach to the lively and active impression of calomel; a cathartic superior to all others, for the purpose of exciting bilious secretion, and of securing thick or consistent evacuations from the bowels."

We do not doubt that emetics are often productive of the benefits here enumerated. But we find in such results no evidence of a primary impression on the mucous membrane of the stomach, by an extraneous poison. If such an irritation possess the power of giving a centripital direction to the blood, engorging the portal circle, and depriving the muscles and skin of their due supply, how can a new irritant, such as tartarized antimony or ipecac. impart a centrifugal tendency, and restore the equilibrium? It should rather augment the very condition it is administered to obviate. Indeed, the beneficial effects of emetic medicines, would seem to us quite irreconcileable with the theory here adopted. But not to cavil on speculative points, let us ask, whether emetics are adapted to all cases of Cholera? Dr. D. seems to make no exception; and yet, we should think, they are by no means invariably proper. When the stomach is loaded, by a previous meal remaining undigested, or when full vomiting has not taken place, an emetic may produce the best effects; but in the absence of the former, and when the latter has occurred, we can see little reason for emetic medicines. Nevertheless, we are willing to grant, that they sometimes revive the functions of the skin, and while we are compelled to believe, that the Professor's recommendation is too indiscriminate and general, we feel it a duty to say, that in the practice of some physicians, they have been too much neglected.

After vomiting, Dr. D. advises calomel, or calomel and opium, and when the system is descending into collapse, brandy in liberal doses has done good. The lancet he condemns as not being "justified either by science or the experience of professional men." It does not, however, appear that he ever employed it during the Epidemic. We are no friends to indiscriminate bleeding, any more than to indiscriminate puking; but we have certainly seen it do much good; and, by no means, understand, that its use is not justified by the experience of the profession.

Of the extent to which our author gave calomel, we are not informed. In one place he speaks of ten grain doses, and we presume him to have been temperate in his mercurial administrations. It would appear, however, that some of our friends in Lexington have given this drug in quantities which are as unprecedented, as the mortality which they sought to avert. We have understood, on pretty good authority, that some patients swallowed not less than a quarter of a pound in two or three days! This reminds us of a letter from a Louisiana correspondent, who, in speaking of the practice in yellow fever, says, "*I drew blood enough to float the General Jackson steam boat, and gave calomel enough to freight her!*"

From being the seat of a distinguished medical school, Lexington is a name of much authority among the physicians of this great valley. Not a county and scarcely a town in all the states south of the Ohio and east of the Mississippi, is without a graduate of the Transylvania school. To all such, the conclusions of its professors and their brethren, are likely to be of paramount influence, in regard to this new, mysterious, and baffling Epidemic. As public journalists, then, whose readers chiefly reside in states which remain to be visited, we have felt it a duty, to refer to the practice of our Lexington friends; and, by placing it in connexion with the desolations of the disease in that city, to warn the profession against too implicit a reliance on the speculations and experience of those, whom, for many just and substantial reasons, they are accustomed to respect.

Of the extent of these desolations, we may judge from the fact, that about a fifteenth part of its population actually perished in the course of two weeks; notwithstanding the comparative absence of poverty, a dense and diversified population, stagnant waters, and public accumulations of filth. But additional evidence of the ravages of the Epidemic have been afforded us by private letters, from one or two of which we shall make such extracts as are adapted to the occasion. On the 13th of June, an intelligent and respectable friend, wrote as follows:

"Our physicians are nearly all *done up*—our stores are all shut—all business at a stand—hotels and taverns closed—printing offices closed—and in short, nothing but apothecaries' shops open. Within the last ten days, it is calculated, that not less than 300 victims have fallen. The *cases* are *numberless*. Nor are they confined to the intemperate, and dissolute, and poverty-stricken. The disease here has taken a more elevated range—many of the most respectable, and wealthy, and temperate have fallen.

"It is true, it has been most severe on the blacks, especially the *free blacks*. But several families of white cittzens have been annihilated, or nearly so. Most of the inhabitants who had the means, have fled—some to be brought back the next day on a bier; some to be buried in the country; and some, probably to escape the disease. For myself, having a large family, and no way to get off, I have remained; and, blessed be God! we are still safe. When I thought of flying, I knew not where to go—the country is full of Cholera. It is raging all around us. So I consoled myself with repeating the words of the Poet—"*Who fights meets death, and death finds him who flies.*" In short, the distress is beyond description! No city police—(at least *visible*)—no board of health—no medical reports—and the streets have for the most part the stillness which pervades the ruins of Palmyra. Such, my dear sir, is the city of Lexington.

"I leave you to imagine the picture of our distress. But I must still add, that the markets are suspended and the bakers' shops shut, with one exception. Not a pound of beef to be got—and very little else. Not even a cracker for sale in the city."

On the 17th, the same gentleman thus expresses himself:—

"It is now calculated—and they are beginning to muster up the names, &c. of the *missing*—that not less than 400 have fallen—and *two thirds of the population absent!* The general suffering has been necessarily great—but cases of *individual* suffering would require volumes. For instance—while Mr. D. and his wife were *both dying*, at midnight, a young gentleman *alone* was attending them, when a loud rap called him to the door. It was a little girl 10 years old, begging him, "for God's sake, to come over the way and help her poor father—he was dying, and she could not get any body to come in." Her poor mother had been buried that afternoon. She returned and had to close his eyes alone. Hundreds of cases, even more affecting than this, might be enumerated. But I forbear. Our physicians say (as perhaps you do) that the disease is *under the control of medicine*, if taken in season—i. e. as I understand it, *before the Cholera sets in.* But, if *Cholera* is so easily cured, please tell me why our learned body of physicians have let 400 slip through their fingers?"

Having suggested, that perhaps some part of the great mortality at Lexington, is referable to a reliance, too exclusive, on certain remedies; and a rejection, not warranted by experience, of others possessing great power, we feel it a duty to say, that the extract just given, seems to disclose a cause of mortal ity entirely different. A panic had seized the inhabitants—they dispersed—they did not properly sustain each other—terror aggravated the disease and rendered medicine unavailing—the first stages of the malady, in which only, it can be successfully managed, were, no doubt, in numerous cases neglected, and when medical men were called in, it is quite obvious they were but poorly sustained. Mr. D. and his wife, respectable inhabitants, advanced in life, and living in the centre of the city—expired with a single person present, and he a young man! On the opposite side of the street, a man sickens and dies, with only his little daughter to nurse him and finally close his eyes! If our correspondent would consider these facts, he might find in them no unsatisfactory answer to his question, why the physicians suffered so many to sink. Wherever the moral courage of the community is greatest, and the inhabitants afford to each other the *earliest* aid, reciprocally inspiring hope and confidence, the epidemic, *cæteris paribus*, will be least destructive. It is impossible too often to repeat these important truths. They should be published far and wide; and by the whole power of the press and the *pulpit*, carried home to the minds and hearts of the entire population. Could this be done in advance of the Epidemic, its shafts would fall harmless around the feet of thousands, otherwise doomed to perish.

---

For several weeks past the northern parts of Kentucky have been ravaged by the Epidemic. It *seems* to have extended from the river, at Maysville, into the interior, and yet it appeared at Lexington and Georgetown, *before* it broke out in Paris and Millersburgh, towns which lie on the great road leading to the ancient metropolis of the state. It is, however, by no means confined to the villages, but invades the most sequestered farm houses, where, in many instances, it has been more fatal, than in the towns. Among other spots it has put forth its power at the Blue Licks and Harrodsburg, old and salubrious watering places; at which not a few were assembled, under the delusive impression, that it would not visit such spots. So far from being safe situations, however, we have understood that the salino-sulphur waters of the Blue Licks, which operate as a cathartic, were found in several cases to be an exciting cause.

In all the towns and villages of the region where it prevails, a flight has been the consequence of its first appearance. The idea of contagion seems to have been uppermost in the public mind; and escape, regarded as the only preventive. Even the medical mind of that district of country, has been mystified by the same phantom; and in several places, the physicians have not only recommended flight to the inhabitants, but have actually flown themselve; leaving those who could not escape, to grapple with the danger as they might! It is with pain we record such occurrences; but they make a part of the history of the Epidemic, and throw much light on the causes of its great mortality, in a tract of country, where from the dryness of the calcarious soil, the abundance of subsistence, the comfortable style of llving, the orderly habits, and the general intelligence of the people, a mitigated visitation might have been reasonably anticipated.

Last summer the same portion of the state was visited by the Epidemic, in the form of a mild diarrhœa, and in autumn a number of deaths occurred in Maysville, Lexington, and other towns; but it was decidedly fatal in Frankfort only; which, it is worthy of remark, remains almost unaffected the present summer, although most of the surrounding villages and farms are sorely afflicted. The same is true, in nearly an equal degree, of Louisville, which suffered much more, last autumn, than during the present spring and summer.

We have said, that the Epidemic seemed to *advance into* Kentucky from the Ohio river; from the same, it has appeared to advance into the State of Ohio. Many of the towns and villages on both banks of the river have been invaded and are now suffering, quite up to Pittsburg, which, although defended by a quarantine battery, 8 or 10 miles below the city, has had a number of cases. At Wheeling, not less than in Maysville, it was more severe and fatal. At Bridgeport, opposite the former, it proved suddenly and dreadfully mortal, though on the first day, almost all the inhabitants fled. Steubenville, Gallipolis and other smaller towns, as New Richmond, 20 miles above Cincinnati, are now suffering more or less. In the rear of all these places, indeed, in the southern portions of the State of Ohio, generally, as in the northern parts of Kentucky, it is daily manifesting itself in some new locality. Thus it seems, like the exhalations of the river, to spread from its valley over the adjoining parts of these two states, and equally, as we understand, over Indiana and Illinois. Indeed the region of country through which the river runs, is that, where, in the west, the disease is now most prevalent; its banks were the first affected, its bed *seems* to be the *nidus* of the pestilence. Thus the spread of Cholera in this country, affords, as it has afforded in all others, much *prima facie* evidence, of contagion—evidence which the credulous think conclusive, and the most philosophical find perplexing.

To this seeming proof of contagious dissemination, we may oppose the unquestionable facts,—that diarrhœa or cholerine is generally prevalent throughout the same region; that fatal cholera has occurred, nearly at the same time, in places remote from each other; that the epidemic distemperature, appeared along the great rivers, on nearly the same days, from the Gulf of Mexico to the mountains; that some villages, at which steam boats have constantly landed, have not yet been attacked; that villages comparatively near to, and in constant intercourse with, towns where the disease prevailed, still remain unaffected, while others more remote are laid waste; as for example, Mayslick, 12 miles from Maysville, on the Lexington road, which remains untouched, while Flemingsburg, eight miles further off, and remote from the great highway, has already suffered to a degree seldom equalled any where; finally, that the experience of every day augments the number of cases, which could not have been contracted by exposure to others laboring under the malady.

Now, if *any* case of Cholera arise, independently of contagion, *every* case may, and from the moment when it is shown, that the disease can be produced without contagion, it is sound medical logic to conclude that no such principle exists. This conclusion, however, must not be regarded as definitive and final, unless it is shown, that a disease, which sometimes originates from some other cause than contagion, can never originate from that cause. Still, it is incumbent on the contagionists to make out every case by special proofs; but in attempting this, they must necessarily labor under this disadvantage—that as the atmosphere is impregnated with a poison which sometimes, at least, produces the disease, it is impossible, in the case in which it follows exposure to a choleric chamber atmosphere, to know whether the disease really arose from that cause. The individual might have been attacked from the other cause if he had not been subject to contact with the sick. In the case of one individual, only, this presumption might always be made; but what shall we say of those well authenticated instances in which several members of a family, and sometimes the visitors, are successively taken down, in places where the disease is not prevailing? There are examples of this kind which, we must acknowledge, are embarrassing to the non-contagionists; but they do not *establish* the existence of contagion—they only render it probable. We shall cite a few not hitherto published.

Dr. B. F. Gard, of this state, has favored us with a letter, from which we make the following extract:

"I send you a notice of a few cases of Epidemic Cholera which occurred in the village of Lockbourne, 10 or 12 miles from Columbus, About the 20th of October, a family was landed from a canal boat directly from Cleaveland. It was reported by the family where they lodged, that the strange gentleman was afflicted with cholera morbus. The facts, however, soon showed that his disease was malignant Cholera, for he passed rapidly into a state of collapse and died in a few hours. On the following day, the wife of the deceased was attacked, and having concealed her situation for some time, medical aid was not obtained until the symptoms became alarming, and she died during the evening.

Three or four days from the death of the strange lady, the landlord and his wife both took the disease; and a poor woman, living three miles distant in the country, having visited the public house for the purpose of rendering some services, was also attacked soon after her return. These cases were treated by Dr. Burrle, of Bloomfield, a gentleman of considerable professional reputation. They all terminated fatally. The three other individuals were slightly attacked; they applied early for medical aid, and were soon restored to health.

It is a remarkable fact, that of all those who attended on the sick, only two escaped an attack of the disease; and, that among the citizens, not a single case occurred except in those who had visited the house above referred to; though there were among them, men of intemperate habits, and those who indulged in a free use of all the fruits of the season. They used no other preventive, than that of avoiding the house where the cholera was known to exist. In speaking of those who attended on the sick, I refer to those cases that terminated fatally, for it does not appear that the disease spread in any of the families where the milder cases occurred, as not more than one was attacked in the same family."

A second example of the same kind, is contained in the following extract of a letter from Dr. Greenleaf Norton, dated Decatur, O., June 26th, 1833.

"I reside in the country, 12 miles north of Maysville. About two weeks ago, midway between this and that town, and near the road leading to it, a man died with but 12 or 18 hours sickness, without having made any application for medical assistance. The day following I learned from his mother, who was with him from the attack to the termination of the disease, the history of the symptoms, which satisfied me, at once, that it was Cholera Spasmodica. I had hitherto, never seen a case of that disease. Two days after his death, his brother sent me word that he had a "lax," and wished me to visit him. When I first saw him, the symptoms were, cold extremities, slight spasms, vomiting, &c. his pulse, though weak, was plainly perceptible. He was out of danger in twelve hours, though not yet entirely recovered.

Being called, two days after, to visit his wife, I saw her at 11, A. M. —she died at 2 P. M. the same day; she was attacked the same morning just before day, with the *formidable* symptoms, having had a diarrhœa 24 hours previous. Her daughter, 12 years of age, was attacked the morning following, at 8 o'clock, and died in 12 or 18 hours. Some 12 or 15 days since, a Mr. D. and son died 12 miles west of this place, of Cholera; as reported by a neighboring physician. A day or two subsequent to this, a Mr. M., son-in-law of D., living six miles west of this, who had, with his wife, visited their sick and dying relatives, was seized with diarrhœa; he took some medicine of his own prescribing; was considerably better, though not free from the "lax," rode about the country for two or three days, when vomiting suddenly commenced with an increase of the diarrhœa, coldness of the extremities, spasms, &c. His wife having had the "lax" for 24 hours, was attacked with vomiting, &c. at the same time with her husband. The mother of Mr. M. a sprightly old lady engaged in nursing them, about 10 o'clock at night began to vomit, and died a little after sunrise the next morning. I have learned since, that she had had diarrhœa for some days."

Much as these cases seem, at first view, to support the doctrine of contagion, we cannot regard them as conclusive. It does not appear that the individuals first attacked, had been exposed to the atmosphere of those who labored under the malady. They were the friends of each other. They were no doubt terrified and grieved. In common with the whole population on both sides of the river for some distance in the country, they were no doubt under the influence of the remote Epidemic power, and their emotions operate as exciting causes.

It has been said by the advocates of flight—the yellow fever is not contagious, and still, there is safety in escaping from the cities where it prevails, why, then, should not the people fly from the towns to the country, to avoid the Cholera? The answer is obvious. The poisonous atmosphere which produces yellow fever, is local, and in general limited to cities:—that which causes Cholera, is general, overspreading both town and country, though in most cases manifesting itself first in the former. How then can we escape the disease by flying from place to place?

We shall conclude this long and desultory article with some account of the Epidemic as it now prevails in Cincinnati.

After its subsidence last fall, the city became healthy, except a moderate prevalence of Scarlatina, which was fatal in a number of cases, though the proportion of our juvenile population attacked by it was small. In some instances it was complicated with Cholera, and proved speedily mortal, with symptoms of collapse. The month of March was dry and healthy. April was unusually dry and hot—so that on the whole it resembled the month of May; which in turn had all the characteristics of the former, being showery and cool throughout, of course with the exception of an occasional hot day. The same remark is applicable to the present month, June, which has been extremely wet, and often so cool as to render fire and winter clothing acceptable. Though there have been much rain and many great and sudden depressions of temperature, there has been less thunder than usual.

On the afternoon of Thursday, the 11th of April, a violent tornado passed over the city and the country north of it, and was followed, as usual, by an exceedingly chilly state of the atmosphere.

On the two succeeding days, we met with several cases of serous diarrhœa and one of cholera morbus, attended with painful cramps of the stomach; which, however, yielded kindly to the usual treatment. But on Sunday morning, the 14th, we saw in consultation, a case of true Epidemic Cholera, which had commenced with copious diarrhœa, the night before, and proved fatal that day. Since then, the disease has prevailed, more or less, in every part of the city; and what is worthy of remark, has had, throughout most of the time, hebdominal exacerbations, much the greatest number of deaths having occurred on Friday, Saturday, Sunday and Monday. We had already made this observation ourselves, when our attention was directed to it by an inteligent medical friend, who felt convinced of its accuracy.

As there was nothing in the habits or pursuits of the people of the city, which could afford an explanation of this fact, we were, like Baron Humboldt, who illustrates the configuration of the mountains and valleys of Mexico, by a reference to the moon, naturally led to direct our attention to that planet, and upon consulting the almanac, which we hereafter advise all reviewers to keep on their tables with their dictionaries and grammars, we found that all the fulls and changes and quarters of the moon, for the succeeding ten weeks from the 11th ot April, occurred on Friday, Saturday, Sunday and Monday. Oi course we sought no further for an explanation of the phenomenon as none more convenient and satisfactory could easily be anticipated.

To what degree the Epidemic was revived by the hurricane of which we have just spoken, we are unable to say. It was more violent to the north of Cincinnati for sixty or seventy miles, than it was here; but it does not appear that the Cholera followed it any where back of the city. How far west or south-west of us it began, or how far it extended to the north-east, we are not informed; but from its width, its length must have been considerable; and if it influenced the reproduction of the Epidemic at this place, it should have had the same effect elsewhere.

From the time when the Epidemic re-appeared in April, to the present, a great number of our citizens have labored under cholerine or diarrhœa—the mild stage of the malignant Cholera. Children, however, have been less affected than adults, and ordinary cases of cholera infantum have been rare. The same is true of the cholera morbus of adults, which has seldom been met with. In most cases there has been a deficiency of bile. The discharges have been often black at first, but have constantly tended to a transparent or whitish character. Bile has very seldom been ejected by vomiting. In many instances the stomach has been sound and the appetite unimpaired, when great increased secretion and peristaltic action existed in the bowels. A number of cases of dysentery have occurred, and were generally more manageable than cases of Cholera. Their symptoms presented nothing uncommon, except that the stomach was more irritable than it usually is in dysentery, and vomiting often supervened. As yet, but few cases of intermitting or remitting fever have occurred. But there has, on the whole, been more febrile action in the early stages of Cholera this spring and summer, than was observed last fall, and the functions of the skin have been less impaired. Most of the cases which have fallen under our observation commenced with diarrhœa, sometimes mild and running on without reducing the patient rapidly, but at other times, profuse and prostrating from the first. In general there has been less cramp and spasm this spring than last autumn.

As to exciting causes, they have not always been obvious. In one person the diarrhœa appeared to be brought on by eating a dozen unripe pears, and the vomiting immediately followed the loss of a dear friend. It proved fatal. In another, grief alone seemed to be the exciting cause. In another, the emotion raised by the departure of a beloved companion, for residence in a distant state, appeared to awaken the malady. In a patient, convalescent from another disease, it was evidently started by the excessive action of a dose of senna, and indulgence in a hearty dinner, *during* the operation. We have heard of several cases, in which it was excited by eating cherries immoderately. No instance of its being produced by intoxication has fallen under our observation, and most of those who have been our patients, have been persons of exemplary sobriety and good habits generally. This fact demands a moment's reflection. A delicate nervous temperament is a great predisposer to Cholera. Persons of this temperament are generally timid, and often circumspect to a fault, both as to the quality and quantity of their food and drinks. In reference to the former, they are not likely to be too particular; but it is quite possible to carry a system of abstemiousness too far. Whatever increases the debility and susceptibility of the system, favors the access of the disease. The firmer the health, and the more vigorous the circulation, provided it be equable, the greater the protection from the Epidemic. Good but regular living, is, then, the proper kind of living, when the Cholera is abroad in the land; and as to drinking, it should, as near as possible, be what each individual is accustomed to, under ordinary circumstances:—care being taken, by those who sink their health, character, fortune, and the happiness of their wives and children in coffee houses, not to carry their drinking to *intoxication*, during the Epidemic. They can indemnity themselves when it has passed away, by getting "dead drunk," oftener than usual, and, thus in the end make up the sum total of their felicity. As the period for new cider is now approaching, it should be recollected, both in town and country, that no drink will be more likely to excite the disease.

The treatment of the Epidemic this spring, has not varied, materially, from that of the preceding autumn. Every day's observation has impressed us more and more deeply, with the great truth already repeated by us more than once—that *to be successful it must be early.* In its first stages, no disease is more amenable to medicine; as it progresses, its obstinacy increases in a higher ratio than that of any other malady—thus neglect is death.

In many cases when the physician is called early, bloodletting is a most valuable remedy. When collapse is approaching, or has become established, the lancet is of little avail, and has sometimes done harm. We have never yet seen the pulse rise after bleeding in any stage of Cholera.

Of emetics we have already expressed ourselves, at large, when speaking of the treatment of the malady in Lexington. We shall only add, in this place, that we have seen several cases, where the gastric irritability was greater after the administration of an emetic than before, but the reverse has sometimes been the result of their operation.

We have not seen the saline practice of Dr. Stevens tried this spring; but a great deal of soda water has been drunk, and apparently with advantage. We have prescribed "weak lye" (extemporaneous solution of the subcarbonate of potash) in a greater number of cases than last fall, and have been much pleased with its effects. We have very often combined paregoric with it. In some cases we have cooled it with ice—at other times administered it as hot as it could be taken. On the whole, we regard it as a valuable remedy, adapted to every stage of the disease.

Opium and internal stimulants, have been used less liberally in the present, than the preceding visitation. The prevailing sentiment of the profession here this spring, has been against them. External irritation, commenced *before* the skin had become cold and bloodless, has zealous advocates, and is undoubtedly deserving of some confidence.

We have given the ethereal tincture of phosphorus, in a few advanced cases. It seemed, in some of them, to moderate the vomiting, but in no instance arrested the disease. We have seen the oil of turpentine administered liberally and perseveringly to one patient in the stage of collapse, but without success.

Enemata of starch, and strong coffee, or decoction of galls, with laudanum, have been repeatedly employed, in our own practice and that of our friends; although they sometimes restrained the diarrhœa, we have seen no case in which they seemed to contribute to a cure. Injections, rendered highly stimulating with powdered mustard, have also been administered, but with no better effect.

Ice and iced water have been used much more extensively than before. They are highly acceptable to all choleric patients, but their effects have not been so salutary as Broussais and other physicians of the continent of Europe, profess to have found them. We have given pellets of ice, repeatedly, to the same patient for 24 hours; but he died at last. We have, also, in a number of cases employed ice externally, especially over the epigastrium and umbilical regions. In one instance, lumps of ice were kept upon those parts for 26 hours, without intermission, during the whole of which time, the skin underneath, was as cold as death; while that of the feet and legs was warm; it was grateful to the patient, and when removed, the irritability of the stomach increased. It evidently did some service; but the patient ultimately died, not, however, in the stage of collapse, but of consecutive fever. On the whole, we regard ice as a valuable remedy, but believe its powers to have been greatly overrated. We have met with no case in which it appeared to do harm.

Calomel seems to have maintained itself in the confidence of the profession, and is given in almost every case. Some physicians administer it in drachm doses; but the greater number limit themselves to 20 or 30 grains, frequently repeated. All the patients, as far as we know, who are salivated recover. Calomel seldom purges in Cholera, and often tranquilizes the bowels; but is sometimes dislodged from the stomach, though when given in powder, it has been supposed to be more difficult of ejection, than any other medicine. In the forming stages of the disease, the blue pill is serviceable. We are strongly impressed with the opinion, that the cure of Cholera, in a great degree depends upon restoring the lost secretory function of the liver. In the first stages, this is easily accomplished; but in the advanced, nothing is more difficult. As an adjuvant to the calomel, we have derived some advantage from the nitrate of potash, which is well calculated to act on the kidneys, while it probably, exerts a beneficial influence on the blood.

The sudorific treatment has been less employed than before; partly, we presume, on account of the greater heat of the weather, in the present than the former visitation. We are disposed to think, that this method is too much neglected. Speculatively, we are even inclined to believe, that if the patient were treated from the beginning with sudorifics, the result would be satisfactory. For instance, suppose, on taking to his bed, he should do nothing but drink an infusion of *Eupatorium perfoliatum* (thoroughwort) impregnated with the alkali of wood ashes, as hot as it could be swallowed, would not a ocpious perspiration at length ensue, and the disease suffer arrest? we are really inclined to recommend this simple method, under the impression, that in all cases in which the functions of the skin are maintained, recovery follows.

To stop the vomiting in the latter stages of the disease, we has given ammoniated alcohol, and lime-water and milk, but with no very satisfactory result. Camphor, in small doses, has sometimes done good. The oxide of bismuth, and musk which especially has been lately extolled, we have not seen used.

We have, thus, given a brief, but comprehensive account of the prevalent treatment of the Epidemic, in its present visitation. In conclusion, we shall add, that for the 10 or 11 weeks which have elapsed, since the Cholera re-appeared among us, it has prevailed in every part of the city, and affected persons of all ranks; but seems to have been less fatal to negroes, compared with whites, than it was in the autumn of 1832. This is, perhaps, conformable to a general law of the Epidemic, by which, in its first visitation, it carries off the greatest proportion of those who are largely exposed to its exciting causes. What number have died is not very accurately known; but it cannot be far from 200; perhaps, something more, or three *per diem;* which is one a day, for every 10,000 inhabitants, a rate of mortality about one-seventh of that of 1832. Then, however, the Epidemic ceased after the fourth week, while it now remains unabated, after nearly three times that period.

